# Small Heat Shock Proteins Potentiate Amyloid Dissolution by Protein Disaggregases from Yeast and Humans

**DOI:** 10.1371/journal.pbio.1001346

**Published:** 2012-06-19

**Authors:** Martin L. Duennwald, AnaLisa Echeverria, James Shorter

**Affiliations:** 1 Boston Biomedical Research Institute, Watertown, Massachusetts, United States of America; 2 Department of Biochemistry and Biophysics, Perelman School of Medicine, University of Pennsylvania, Philadelphia, Pennsylvania, United States of America; Brandeis University, United States of America

## Abstract

How small heat shock proteins (sHsps) might empower proteostasis networks to control beneficial prions or disassemble pathological amyloid is unknown. Here, we establish that yeast sHsps, Hsp26 and Hsp42, inhibit prionogenesis by the [*PSI* ^+^] prion protein, Sup35, via distinct and synergistic mechanisms. Hsp42 prevents conformational rearrangements within molten oligomers that enable *de novo* prionogenesis and collaborates with Hsp70 to attenuate self-templating. By contrast, Hsp26 inhibits self-templating upon binding assembled prions. sHsp binding destabilizes Sup35 prions and promotes their disaggregation by Hsp104, Hsp70, and Hsp40. In yeast, Hsp26 or Hsp42 overexpression prevents [*PSI^+^*] induction, cures [*PSI^+^*], and potentiates [*PSI^+^*]-curing by Hsp104 overexpression. In vitro, sHsps enhance Hsp104-catalyzed disaggregation of pathological amyloid forms of α-synuclein and polyglutamine. Unexpectedly, in the absence of Hsp104, sHsps promote an unprecedented, gradual depolymerization of Sup35 prions by Hsp110, Hsp70, and Hsp40. This unanticipated amyloid-depolymerase activity is conserved from yeast to humans, which lack Hsp104 orthologues. A human sHsp, HspB5, stimulates depolymerization of α-synuclein amyloid by human Hsp110, Hsp70, and Hsp40. Thus, we elucidate a heretofore-unrecognized human amyloid-depolymerase system that could have applications in various neurodegenerative disorders.

## Abstract 2

The authors define how small heat-shock proteins synergize to regulate the assembly and disassembly of a beneficial prion, and then they exploit this knowledge to identify the human amyloid depolymerase.

## Author Summary

Amyloid fibers are protein aggregates that are associated with numerous neurodegenerative diseases, including Parkinson's disease, for which there are no effective treatments. They can also play beneficial roles; in yeast, for example, they are associated with increased survival and the evolution of new traits. Amyloid fibers are also central to many revolutionary concepts and important questions in biology and nanotechnology, including long-term memory formation and versatile self-organizing nanostructures. Thus, there is an urgent need to understand how we can promote beneficial amyloid assembly, or reverse pathogenic assembly, at will. In this study, we define the mechanisms by which small heat-shock proteins synergize to regulate the assembly and disassembly of a beneficial yeast prion. We then exploit this knowledge to discover an amyloid depolymerase machinery that is conserved from yeast to humans. Remarkably, the human small heat shock protein, HspB5, stimulates Hsp110, Hsp70, and Hsp40 chaperones to gradually depolymerize amyloid fibers formed by α-synuclein (which are implicated in Parkinson's disease) from their ends on a biologically relevant timescale. This newly identified and highly conserved amyloid-depolymerase system could have important therapeutic applications for various neurodegenerative disorders.

## Introduction

Amyloid fibers are thread-like protein polymers with cross-β structure. These unusually stable, self-templating structures were first identified in various systemic amyloidoses and neurodegenerative disorders such as Alzheimer's disease [1]. In isolation, many proteins can form amyloid fibers, suggesting that amyloidogenesis is an intrinsic property of polypeptides [1]–[3]. Indeed, amyloid conformers have been captured during evolution for various beneficial purposes, including prion-based transmission of advantageous phenotypes, long-term memory formation, melanosome biogenesis, drug resistance, and biofilm formation [4]–[14]. Moreover, as stable self-organizing polymers, amyloids are interesting nanomaterials [15]–[19]. Thus, in diverse fields there is an urgent need to understand how we can promote or reverse amyloidogenesis as necessary.

We hypothesized that small heat shock proteins (sHsps) might enable control of amyloidogenic trajectories. sHsps are the most widespread family of molecular chaperones [20],[21]. sHsps protect cells from diverse environmental stresses by suppressing amorphous aggregation of denatured proteins [20]–[23]. All sHsps harbor a conserved C-terminal α-crystallin domain of ∼90 residues, but are otherwise diverse in size and sequence. Typically, sHsps form large dynamic oligomers and function as ATP-independent chaperones that bind denatured proteins to prevent aggregation [20]–[23]. sHsps maintain proteins in a soluble form that can be reactivated by Hsp70 [24]–[26].

The yeast cytosol harbors two sHsps: Hsp42 and Hsp26. Both form large dynamic oligomers of 24 subunits [27]–[30]. Hsp42 is more abundant and prevents protein aggregation at physiological and heat shock temperatures [28]. By contrast, Hsp26 is activated as a chaperone at elevated temperatures via complex changes in the quaternary dynamics of its oligomer [27],[29],[31],[32]. Hsp26 and Hsp42 display overlapping and broad substrate specificity [28]. Incorporation of Hsp26 into denatured aggregates can promote their dissolution and renaturation by Hsp104 and Hsp70 [26],[33]. However, if Hsp26 is added to preformed denatured aggregates, then it cannot assist Hsp104 and Hsp70 [33]. Much less is known about Hsp42, which might be involved in aggregate partitioning in vivo [34]. Whether Hsp42 interacts directly with Hsp104 or Hsp70 is unknown.

Despite these advances in understanding how sHsps handle denatured proteins, much less is known about how sHsps might interface with amyloidogenic folding pathways. sHsps might inhibit different steps in amyloidogenesis of various disease proteins, such as α-synuclein (α-syn), polyglutamine, or Aβ40 [23],[33],[35]–[38]. Yet it is unknown whether sHsps enable the proteostasis machinery to disassemble pathological amyloid. Furthermore, it is unknown whether sHsps regulate beneficial amyloid.

**Figure 1:**
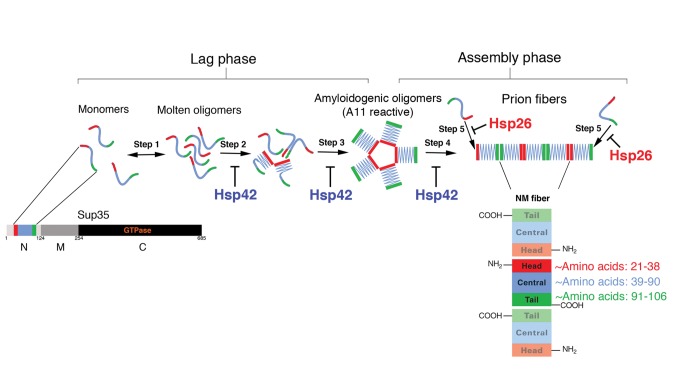
Mechanism of Sup35 prion assembly. Sup35 is composed of a C-terminal GTPase domain (amino acids 254–685, black) that confers translation termination activity, a highly charged middle domain (M, amino acids 124–253, dark grey), and a prionogenic N-terminal domain (N, amino acids 1–123, light grey) enriched in glutamine, asparagine, tyrosine, and glycine. Together N and M (NM) confer all the properties needed to form a stable prion in yeast [94]. Hence, NM is termed the prion domain. Within N, prion recognition elements termed the “Head” (red) and “Tail” (green), which flank a “Central Core” (blue), play important roles in prionogenesis. After a lag phase (steps 1–3), Sup35 prions assemble rapidly (steps 4 and 5). Prion recognition elements within N make homotypic intermolecular contacts such that Sup35 prions are maintained by an alternating sequence of Head-to-Head (red) and Tail-to-Tail (green) contacts. The Central Core (blue) is sequestered by intramolecular contacts. The amino acids that comprise the Head, Core, and Tail region when NM is assembled at 25°C are indicated. The steps antagonized by Hsp26 and Hsp42 are indicated.

In this study, we address these issues by first employing the yeast translation termination factor, Sup35 (Figure 1). Sup35 forms infectious amyloids (prions) that transmit heritable reductions in translation termination fidelity and comprise the yeast prion [*PSI^+^*] [8]. [*PSI^+^*]-encoded reductions in translation termination fidelity confer phenotypic diversity and selective advantages to yeast in diverse environments [8],[10],[11],[13], but can be deleterious in other settings [39]. Sup35 is a valuable paradigm for studying prion-folding events, with analytical tools that are unavailable for other amyloids or prions. Indeed, these tools have helped clarify how Sup35 prions assemble [40],[41]. They have also revealed various aspects of Sup35 prion structure at the resolution of spatial arrangements of individual amino acids involved in inter- and intra-molecular contacts (Figure 1) [40]–[42]. These tools provide unique opportunities to establish a detailed understanding of how sHsps affect prion assembly and disassembly.

The two N-terminal domains of Sup35, termed NM, confer all the properties needed to form a stable prion in yeast (Figure 1) [8]. In isolation, NM spontaneously forms prions by a well-defined mechanism that involves a lag phase and assembly phase [40],[43],[44]. Early in lag phase, NM partitions between a monomeric (∼90% total NM) and oligomeric (∼10% total NM) pool (Figure 1, step 1) [40],[43],[45],[46]. NM monomers are largely unstructured and populate multiple transient conformations [47]. However, the specific intermolecular contacts required for prion formation ultimately form in molten NM oligomers. NM monomers within structurally fluid oligomers gradually reorganize (Figure 1, step 2) to form amyloidogenic oligomers (Figure 1, step 3), which are structurally distinct to fibers [40],[43],[48],[49]. The intermolecular contacts that define prions form very rapidly once these obligate, transient intermediates appear (Figure 1, step 4) [40]–[42],[48],[49]. Amyloid fibers then seed their own rapid bidirectional assembly by capturing and converting monomers to the cross-β form (Figure 1, step 5) [43],[46],[50]. Short prion recognition elements within the N-terminal domain (N), termed the “Head” and “Tail”, are proposed to make homotypic intermolecular contacts in assembled prions [40]–[42],[51],[52]. Thus, prions are maintained by alternating Head-to-Head and Tail-to-Tail contacts that separate a central core (Figure 1). Both the Head and Tail regions can nucleate prion assembly, although the rate-limiting step of lag phase is the establishment of the Head-to-Head contact [40]–[42],[51]. This well-defined sequence of prion-folding events provides an unparalleled opportunity to understand how sHsps affect prion formation at a molecular level.

How Hsp26 and Hsp42 might affect prion-folding events in yeast is unclear. Both Hsp26 and Hsp42 are found to be associated with ex vivo SDS-resistant prion aggregates [53], but deletion of Hsp26 does not affect [*PSI^+^*] propagation [12] and overexpression of Hsp26 or Hsp42 does not cure [*RNQ^+^*] [33]. However, beyond these observations nothing is known about how these sHsps might affect prion-folding events. It is also unclear whether sHsps contribute to the dissolution of amyloid or prion conformers by the proteostasis network. In yeast, the protein disaggregase and AAA+ ATPase, Hsp104, can rapidly disassemble amyloid conformers [48],[49],[54]–[58]. Overexpression of Hsp26 or Hsp42 together with Hsp104 can increase soluble levels of polyglutamine in yeast, but whether this reflected enhanced disaggregation or inhibition of aggregation remains unknown [33]. Curiously, metazoan proteostasis networks lack an Hsp104 homologue [59]. Thus, it is unclear how amyloid dissolution is catalyzed in these systems [60]. We have recently defined a mammalian disaggregase machinery composed of Hsp110 (Apg-2), Hsp70 (Hsc70 or Hsp70), and Hsp40 (Hdj1), which resolves denatured aggregates, but does not rapidly remodel amyloid [61]. Yet amyloid fibers are dynamic entities and monomers at fiber ends can slowly dissociate and rapidly reassociate [62]–[68]. Whether proteostasis networks capitalize on this molecular recycling to promote gradual amyloid depolymerization is unknown. Here, we define how sHsps regulate beneficial Sup35 prions and potentiate amyloid dissolution.

## Results

### Hsp26 and Hsp42 Synergize to Inhibit Spontaneous Sup35 Prionogenesis

**Figure 2:**
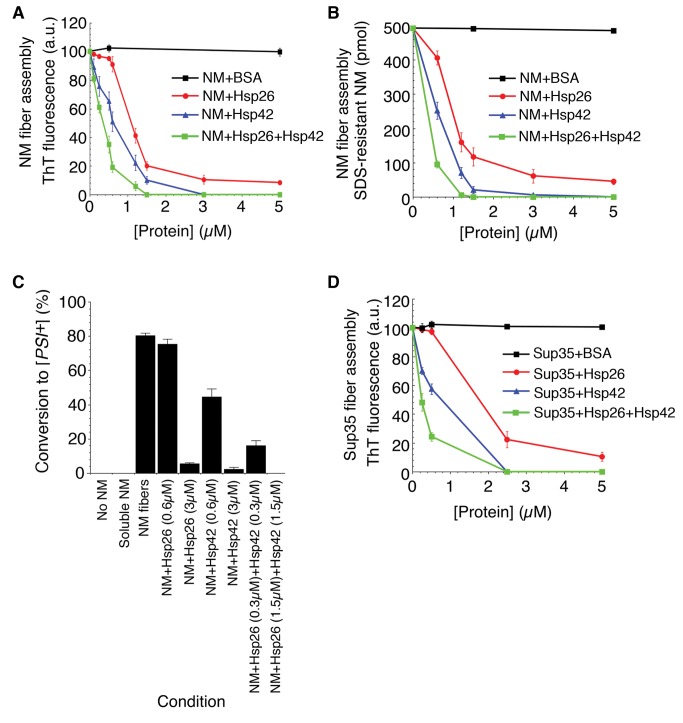
Hsp26 and Hsp42 synergize to inhibit spontaneous Sup35 prionogenesis. (A, B) NM (5 µM) was incubated at 25°C with agitation for 6 h in the presence of increasing concentrations of BSA, Hsp26, Hsp42, or Hsp26 and Hsp42 (0–5 µM). For the mixture of Hsp26 and Hsp42, a 1∶1 ratio was employed. Thus, a concentration of 2 µM on the *x*-axis reflects 1 µM Hsp26 and 1 µM Hsp42. Fibrillization was measured by Thioflavin-T (ThT) fluorescence (A) or by determining the amount of SDS-resistant NM (B). Values represent means±SD (*n* = 3). (C) NM (5 µM) was assembled at 25°C with agitation for 6 h in the absence or presence of Hsp26 (0.6 µM or 3 µM), Hsp42 (0.6 µM or 3 µM), or Hsp26 and Hsp42 (0.3 µM or 1.5 µM of each). Reaction products were concentrated and transformed into [*psi* ^−^] cells. No NM and soluble NM served as negative controls. The proportion of [*PSI* ^+^] colonies was then determined. Values represent means±SD (*n* = 3). (D) Sup35 (5 µM) was incubated at 25°C with agitation for 6 h in the presence of increasing concentrations of BSA, Hsp26, Hsp42, or Hsp26 and Hsp42 (0–5 µM). For the mixture of Hsp26 and Hsp42, a 1∶1 ratio was employed. Thus, a concentration of 2 µM on the *x*-axis reflects 1 µM Hsp26 and 1 µM Hsp42. Fibrillization was measured by ThT fluorescence. Values represent means±SD (*n* = 3).

Using complementary methods, including Thioflavin-T (ThT) fluorescence and SDS-resistance, we demonstrated that Hsp42 potently inhibited (IC_50_∼0.67 µM of Hsp42 monomer) spontaneous NM fibrillization (Figure 2A,B blue markers). Marked inhibition was observed at a ratio of NM∶Hsp42 of 10∶1 and assembly was abolished at an NM∶Hsp42 ratio of 1.67∶1 (Figure 2A,B). Hsp26 also inhibited (IC_50_∼1.1 µM) spontaneous NM fibrillization (Figure 2A,B red markers). Hsp26 was not as effective as Hsp42, but inhibition was observed at a ratio of NM∶Hsp26 of 4∶1 (Figure 2A,B). A 5-fold molar excess of Hsp26 was needed to completely block NM fibrillization. These inhibitory effects truly precluded prion formation because NM incubated in the presence of sHsps failed to transform [*psi* ^−^] cells to [*PSI^+^*] (Figure 2C). Typically, sHsps bind 1 substrate per ∼2–3 sHsp monomers [24],[27]. Thus, the strong inhibition at substoichiometric concentrations indicates that the sHsps might inhibit a rare or transient NM conformer that is critical for prion formation.

Remarkably, the inhibitory activities of Hsp26 and Hsp42 were synergistic. The combination of an equimolar mixture of Hsp26 and Hsp42 was a more potent inhibitor (IC_50_∼0.16 µM, i.e. 0.08 µM of each sHsp) of NM assembly than either sHsp alone (Figure 2A–C). Hsp26 and Hsp42 also synergized to inhibit spontaneous prionogenesis of full-length Sup35 (Figure 2D). To the best of our knowledge, this is the first example of two distinct sHsps working together in a synergistic manner to prevent prion formation.

### Hsp26 and Hsp42 Inhibit De Novo Sup35 Prionogenesis by Distinct Mechanisms

**Figure 3:**
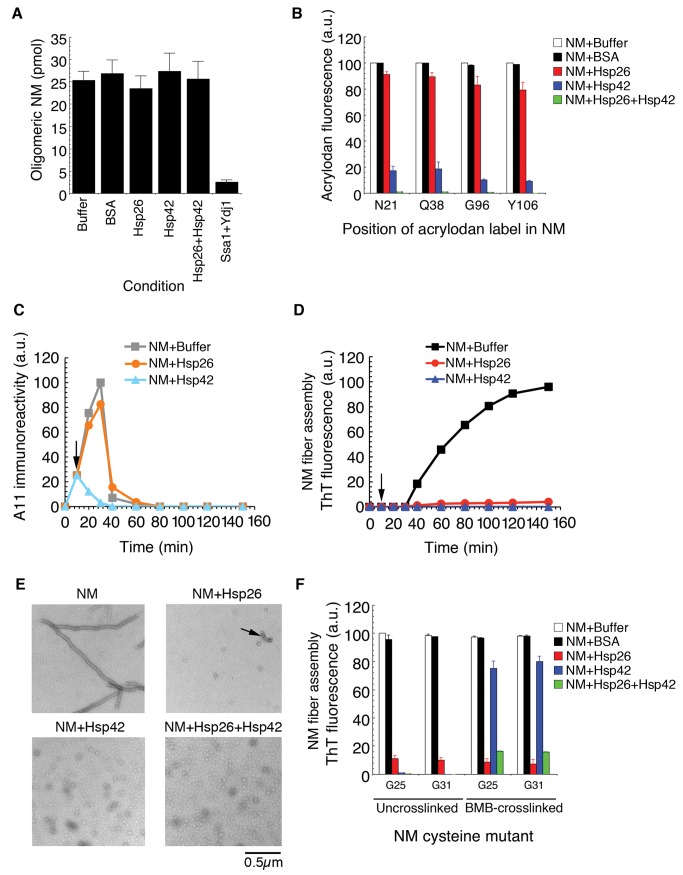
Hsp26 and Hsp42 inhibit de novo Sup35 prionogenesis by distinct mechanisms. (A) NM (5 µM) was rotated for 5 min (80 rpm) in the absence or presence of Hsp26 (3 µM), Hsp42 (3 µM), or Ssa1 plus Ydj1 (3 µM). Oligomeric NM was recovered by centrifugation at 436,000 g for 30 min, resolved by SDS-PAGE, Coomassie stained, and the amount in the pellet fraction determined. Values represent means±SD (*n* = 3). (B) Fluorescence of NM-N21C-, Q38C-, G96C-, or Y106C-acrylodan (5 µM) after 15 min at 25°C in the absence or presence of BSA (3 µM), Hsp26 (3 µM), Hsp42 (3 µM), or Hsp26 (1.5 µM) and Hsp42 (1.5 µM). Values represent means±SD (*n* = 3). (C, D) NM (5 µM) was incubated at 25°C with agitation for 10 min at which point (arrow) buffer, Hsp26 (3 µM), or Hsp42 (3 µM) was added. The reaction was then continued at 25°C with agitation to 150 min. At the indicated times, the amount of A11-reactive species present was determined (C) or the amount of ThT-reactive species was determined (D). Datasets representative of three replicates are shown. (E) Electron microscopy of NM assembly at 25°C with agitation for 6 h in the absence or presence of Hsp26 (3 µM), Hsp42 (3 µM), or Hsp26 (1.5 µM) and Hsp42 (1.5 µM). Note the presence of small fibers in the presence of Hsp26 (arrow), the accumulation of oligomers in the presence of Hsp42 or Hsp26 and Hsp42. Bar, 0.5 µm. (F) NM cysteine variants were either left uncrosslinked or crosslinked under denaturing conditions with a flexible 11 Å BMB crosslink at position 25 or 31. The indicated NM protein (5 µM) was then assembled with agitation at 25°C in the absence or presence of BSA (3 µM), Hsp26 (3 µM), Hsp42 (3 µM), or Hsp26 and Hsp42 (1.5 µM of each). Fibrillization was measured by ThT fluorescence. Values represent means±SD (*n* = 3).

The synergistic inhibition of Sup35 prionogenesis by Hsp26 and Hsp42 suggested that the two sHsps might inhibit prion formation by distinct mechanisms. The ability of the sHsps to abrogate prion formation at substoichiometric concentrations also suggested interference with a specific conformer or intermediate that is initially present at low concentrations. Two non-mutually exclusive possibilities emerge. First, sHsps might mask the ends of *de novo* formed NM fibers and thus prevent seeded assembly (Figure 1, step 5). Second, sHsps might antagonize the formation (Figure 1, step 1) or reorganization (Figure 1, steps 2–4) of transient molten oligomers. Only a small fraction (∼10%) of the total NM accesses molten oligomeric forms [45], which are obligate reaction intermediates for spontaneous fibrillization (Figure 1) [48],[49]. These malleable NM oligomers possess a hydrodynamic radius of 50–130 nm, form extremely rapidly, and can be recovered by ultracentrifugation [45],[55]. Importantly, neither Hsp26 nor Hsp42 alone or in combination inhibited the formation (Figure 1, step 1) of NM oligomers (Figure 3A). By contrast, the combination of Ssa1 (an Hsp70) and Ydj1 (an Hsp40) inhibited oligomer formation (Figure 3A) [55].

After their initial formation, molten NM oligomers gradually reorganize into amyloidogenic forms (Figure 1, steps 2 and 3) that ultimately elicit assembly phase (Figure 1, step 4). This maturation process can be tracked using single cysteine NM mutants labeled with acrylodan at specific positions [40],[41]. Sequestration of labeled sites from solvent yields increases in acrylodan fluorescence. Specific portions of N (∼residues 21–106) gradually become solvent inaccessible in molten oligomers prior to fiber assembly (Figure 1, steps 2 and 3). This process begins immediately and the maximal increase in acrylodan fluorescence signals the end of lag phase and the start of assembly phase [40]. To determine whether Hsp26 or Hsp42 interfered with this process we utilized NM with acrylodan attached to cysteines replacing Asn21, Gln38, Gly96, or Tyr106. These mutated and labeled NM variants retain wild-type assembly kinetics and ability to access infectious forms [40]–[42]. We measured acrylodan fluorescence after 15 min, when the assembly reaction remained in lag phase (Figure 3B). Hsp26 had only a slight inhibitory effect on increases in acrylodan fluorescence at all positions tested, suggesting that Hsp26 does not interfere with oligomer maturation (Figure 3B; Figure 1, steps 2 and 3). By contrast, Hsp42 inhibited increases in acrylodan fluorescence at all positions tested (Figure 3B). We conclude that Hsp42 inhibits spontaneous NM fibrillization by preventing the maturation of molten NM oligomers (Figure 1, steps 2 and 3). Remarkably, Hsp26 and Hsp42 together caused the greatest inhibition of increased acrylodan fluorescence (Figure 3B).

Next, we determined how Hsp42 or Hsp26 affected an obligate on-pathway oligomeric intermediate in spontaneous Sup35 assembly, which accumulates during lag phase and is specifically detected by the conformation-specific antibody A11 [48],[49],[69]. This oligomeric species is most abundant at the end of lag phase and then rapidly disappears during assembly phase (Figure 1) [48],[49]. We performed a kinetic experiment where NM assembly was initiated for 10 min at which time either buffer, Hsp26, or Hsp42 were added (arrow in Figure 3C,D) and the reaction was then allowed to continue. After 10 min, A11-reactive species had already accumulated (Figure 3C), whereas no fibers had assembled as determined by the lack of ThT fluorescence (Figure 3D). Addition of buffer after 10 min had no effect on assembly and A11-reactive species continued to accumulate until the end of lag phase (∼30 min, Figure 3C, grey markers). A11-reactive species then rapidly declined as fiber assembly initiated (∼40 min, compare grey markers in Figure 3C to black markers in Figure 3D). Addition of Hsp42 after 10 min caused a rapid disappearance of A11-reactive species (Figure 3C, cyan markers) and fiber assembly was blocked (Figure 3D, blue markers). Thus, Hsp42 reversed the formation of A11-reactive conformers.

By contrast, addition of Hsp26 had no effect on the accumulation of A11-reactive species during lag phase or their rapid decline after 40 min (Figure 3C, orange markers). Yet very little fiber assembly occurred (Figure 3D, red markers). Thus, in contrast to Hsp42, Hsp26 does not inhibit or reverse oligomer maturation events (Figure 1, steps 1–4). Rather, these data suggest that Hsp26 inhibits the growth of newly formed fibers (Figure 1, step 5). Indeed, electron microscopy revealed that a few very short fibers assembled in the presence of Hsp26 (arrow in Figure 3E). By contrast, oligomeric structures persisted in the presence of Hsp42, or Hsp26 and Hsp42 (Figure 3E).

To pinpoint which steps of Sup35 prionogenesis are antagonized by Hsp42 or Hsp26, we experimentally bypassed the requirement for oligomer maturation in spontaneous prion formation (Figure 1, steps 2–4). Thus, we crosslinked single cysteine NM mutants in specific positions with the flexible 11 Å crosslinker: 1,4-bis-maleimidobutane (BMB). NM that is BMB-crosslinked at cysteines at position Gly25 or Gly31 assembles into fibers without a detectable lag phase [40],[41]. Hsp26 or Hsp42 potently inhibited the assembly of uncrosslinked NM, and the combination of Hsp26 and Hsp42 was the most effective (Figure 3F). By contrast, Hsp26 but not Hsp42 inhibited assembly of NM that was BMB-crosslinked at position 25 or 31 (Figure 3F). The combination of Hsp26 and Hsp42 was less effective against NM that had been BMB-crosslinked at position 25 or 31 (Figure 3F). These data suggest that Hsp26 selectively antagonizes events after lag phase (Figure 1, step 5), whereas Hsp42 selectively antagonizes oligomer-remodeling events during lag phase (Figure 1, steps 2–4). Thus, Hsp26 antagonizes events that occur after prion recognition elements have initially formed intermolecular contacts, whereas Hsp42 prevents initial formation of these contacts. These data suggest that Hsp26 and Hsp42 synergize to directly antagonize Sup35 prionogenesis prior to intermolecular contact formation by prion recognition elements.

### Hsp26 and Hsp42 Inhibit Sup35 Prion Formation In Vivo

**Figure 4:**
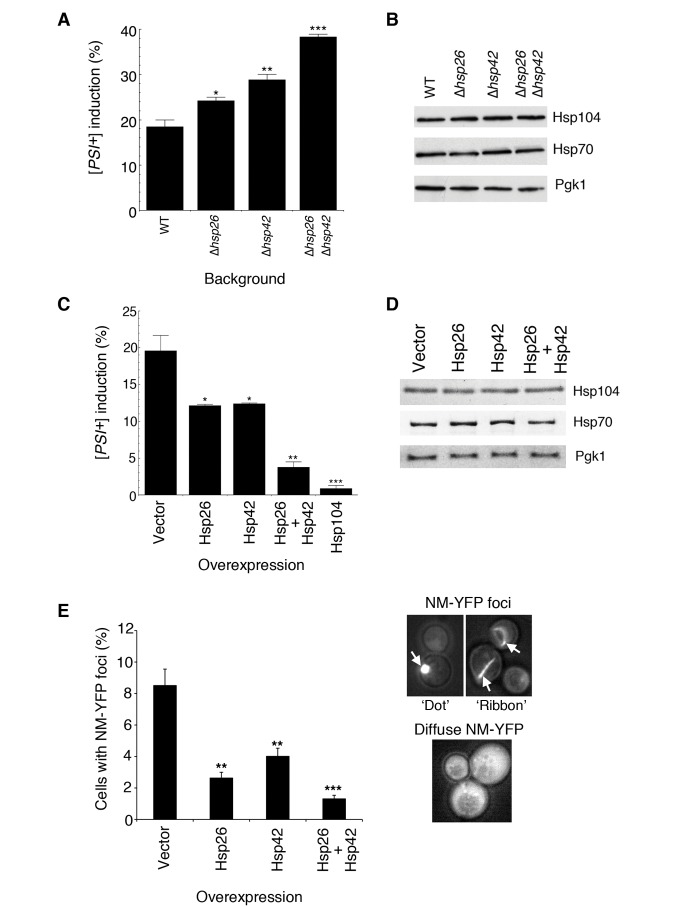
Hsp26 and Hsp42 antagonize Sup35 prion formation in vivo. (A) Sup35 was overexpressed for 16 h at 30°C in wild-type, Δ*hsp26*, *Δhsp42*, or Δ*hsp26*Δ*hsp42* [*psi* ^−^] [*RNQ^+^*] cells. Cells were plated on 25% YPD and the proportion of [*PSI^+^*] colonies was determined. Compared to wild-type cells there was significantly more [*PSI^+^*] induction in Δ*hsp26* cells (*p = 0.0264, two-tailed Student's *t* test), Δ*hsp42* cells (**p = 0.0059, two-tailed Student's *t* test), or Δ*hsp26*Δ*hsp42* cells (***p = 0.0002, two-tailed Student's *t* test). Values represent means±SEM (*n* = 3). (B) Immunoblots demonstrating that neither Hsp104 nor Hsp70 (3A3 antibody that recognizes yeast Ssa1, Ssa2, Ssa3, and Ssa4 [95]) expression is affected in the Δ*hsp26*, *Δhsp42*, or Δ*hsp26*Δ*hsp42* background. Pgk1 is included as a loading control. (C) Sup35 was overexpressed for 16 h at 30°C in [*psi* ^−^] [*RNQ^+^*] cells expressing elevated levels of Hsp26, Hsp42, Hsp26 plus Hsp42, or Hsp104. Cells were plated on 25% YPD and the proportion of [*PSI^+^*] colonies was determined. Compared to the vector control there was significantly less [*PSI^+^*] induction in cells expressing Hsp26 (*p = 0.0253, two-tailed Student's *t* test), Hsp42 (*p = 0.028, two-tailed Student's *t* test), Hsp26 and Hsp42 (**p = 0.0021, two-tailed Student's *t* test), or Hsp104 (***p<0.0001, two-tailed Student's *t* test). Values represent means±SEM (*n* = 3). (D) Immunoblots demonstrating that neither Hsp104 nor Hsp70 (3A3 antibody that recognizes yeast Ssa1, Ssa2, Ssa3, and Ssa4 [95]) expression is affected by elevated expression of Hsp26, Hsp42, or Hsp26 and Hsp42. Pgk1 is included as a loading control. (E) NM-YFP was transiently overexpressed for 4 h at 30°C in [*psi* ^−^] [*RNQ^+^*] cells expressing elevated levels of Hsp26, Hsp42, or Hsp26 and Hsp42. Cells were processed for fluorescence microscopy. The proportion of cells with NM-YFP foci was then determined. (**p<0.01, ***p<0.001, two-tailed Student's *t* test). Values represent means±SEM (*n* = 3). Examples of cells with NM-YFP foci (“dots” and “ribbons” indicated by arrows) and cells with diffuse NM-YFP fluorescence are shown on the right.

These findings suggested that Hsp26 and Hsp42 might antagonize [*PSI^+^*] induction in vivo. Prion nucleation involves protein–protein interactions [40],[43]. Thus, [*PSI^+^*] induction frequency is very low unless Sup35 or NM is overexpressed [70],[71]. Indeed, when Sup35 was transiently overexpressed, [*PSI^+^*] induction increased from barely detectable levels (<1 in 1,000) to ∼20% of cells (Figure 4A). [*PSI^+^*] induction was modestly increased by ∼1.3-fold in Δ*hsp26* cells and ∼1.6-fold in Δ*hsp42* cells (Figure 4A). In a double deletion Δ*hsp26*Δ*hsp42* strain, [*PSI^+^*] induction was increased by ∼2.1-fold (Figure 4A). Importantly, immunoblots revealed that neither Hsp104 nor Hsp70 expression were affected by the sHsp deletion (Figure 4B). Thus, increased [*PSI^+^*] induction observed in the sHsp deletion strains (Figure 4A) is likely to be a direct effect of reduced sHsp activity.

Elevated expression of Hsp26 or Hsp42 antagonized [*PSI^+^*] induction by Sup35 overexpression (Figure 4C). The combination of Hsp26 and Hsp42 was more effective than either sHsp alone, and almost as effective as the protein disaggregase Hsp104 (Figure 4C). The expression of Hsp104 and Hsp70 was not altered by sHsp overexpression (Figure 4D), indicating that these effects are likely due to sHsp activity. Accordingly, Hsp26 and Hsp42 overexpression prevented the formation of NM-YFP foci, a reporter of [*PSI^+^*] induction, when NM-YFP was overexpressed (Figure 4E) [72]. Here too, the combination of Hsp26 and Hsp42 yielded the greatest inhibition (Figure 4E). Importantly, overexpression of Hsp26, Hsp42, or both has no effect on the [*RNQ^+^*] prion [33], which is critical for [*PSI* ^+^] induction by Sup35 overexpression [73]. Taken together, these data suggest that Hsp26 and Hsp42 work together to directly antagonize [*PSI^+^*] induction.

### Hsp26 and Hsp42 Prevent “Cross-Seeding” by Rnq1 Prions

**Figure 5:**
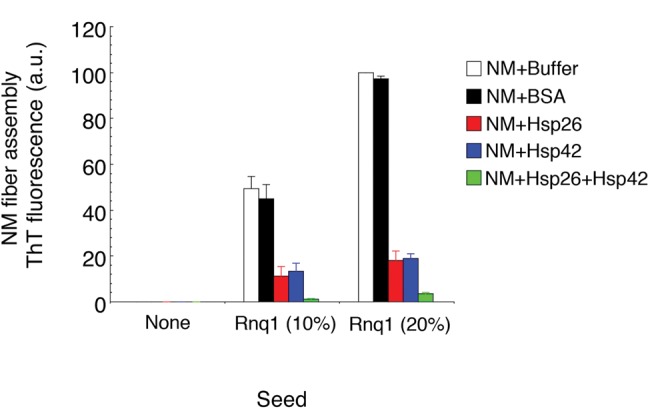
Hsp26 and Hsp42 inhibit cross-seeding by Rnq1 fibers. NM (2 µM) was incubated at 25°C for 16 h with or without Rnq1 fibers (10% or 20% wt/wt) without agitation in the absence or presence of BSA (3 µM), Hsp26 (3 µM), Hsp42 (3 µM), or Hsp26 (1.5 µM) and Hsp42 (1.5 µM). Fibrillization was measured by ThT fluorescence. Values represent means±SD (*n* = 3).

The induction of [*PSI* ^+^] by Sup35 overexpression depends on the presence of another prion [*RNQ^+^*], which is comprised of infectious Rnq1 amyloid [73]. Rnq1 prions are proposed to template the initial formation of Sup35 prions in vivo, and this activity has been reconstituted in vitro [74]. Thus, we tested whether Hsp26 and Hsp42 inhibited NM fibrillization cross-seeded by Rnq1 fibers in vitro. Hsp26 or Hsp42 inhibited NM assembly that was cross-seeded by Rnq1 fibers, whereas a control protein (BSA) had no effect (Figure 5). The ability of Hsp42 to inhibit this seeding reaction was unexpected and might suggest that Rnq1 fibers accelerate events in the lag phase of NM assembly (Figure 1, steps 1–4), rather than acting as a direct template for NM fibrillization (Figure 1, step 5) [74]. Hsp26 and Hsp42 might interact directly with Rnq1 fibers to prevent interactions with NM. Alternatively, interactions between Hsp26 or Hsp42 and NM might prevent interactions with Rnq1 that drive cross-seeding. Importantly, the combination of Hsp26 and Hsp42 was more potent than either sHsp alone (Figure 5), suggesting that the two sHsps work together to prevent cross-seeding. Thus, Hsp26 and Hsp42 antagonize *de novo* formation of Sup35 prions in vitro and in vivo.

### Hsp26 Inhibits Seeded Assembly More Potently Than Hsp42

**Figure 6:**
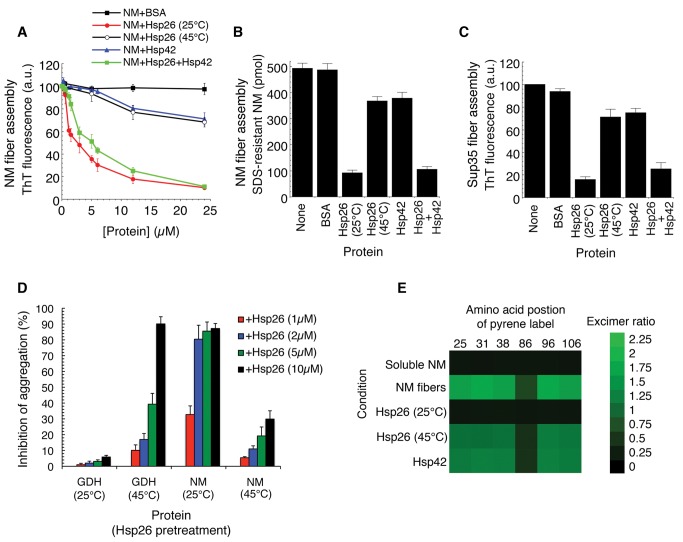
Hsp26 inhibits seeded assembly of Sup35 more potently than Hsp42 in a temperature-sensitive manner. (A) NM (5 µM) was incubated at 25°C for 12 h in the presence of preformed NM fibers (5% wt/wt) plus increasing concentrations of either BSA, Hsp26, Hsp42, or Hsp26 and Hsp42 (0–24 µM). For the mixture of Hsp26 and Hsp42, a 1∶1 ratio was employed. Thus, a concentration of 2 µM on the *x*-axis reflects 1 µM Hsp26 and 1 µM Hsp42. For the Hsp26 alone condition, Hsp26 was pretreated at either 25°C or 45°C for 10 min. Fibrillization was measured by ThT fluorescence. Values represent means±SD (*n* = 3). (B) NM (5 µM) was incubated at 25°C for 12 h in the presence of preformed NM fibers (5% wt/wt) plus BSA, Hsp26, or Hsp42 (12 µM). Hsp26 was pretreated at either 25°C or 45°C for 10 min. Fibrillization was measured by determining the amount of SDS-resistant NM. Values represent means±SD (*n* = 3). (C) Sup35 (5 µM) was incubated at 25°C for 12 h in the presence of preformed Sup35 fibers (5% wt/wt) plus BSA, Hsp26, or Hsp42 (12 µM). Hsp26 was pretreated at either 25°C or 45°C for 10 min. Fibrillization was measured by ThT fluorescence. Values represent means±SD (*n* = 3). (D) Chemically denatured GDH or NM (5 µM) was incubated at 25°C for 4 h with agitation in the presence of Hsp26 (1–10 µM), which had been pretreated at either 25°C or 45°C for 10 min. GDH aggregation was assessed by turbidity and NM fibrillization by ThT fluorescence. Values represent means±SD (*n* = 3). (E) NM proteins (5 µM) carrying pyrene labels at the indicated single site were assembled at 25°C for 12 h in the presence of preformed NM fibers (5% wt/wt) plus either Hsp26 or Hsp42 (12 µM). Hsp26 was pretreated at either 25°C or 45°C for 10 min. The ratio of excimer to non-excimer fluorescence (I_465 nm_/I_375 nm_) was then determined as a measure of intermolecular contact formation. Soluble NM serves as a negative control.

The ability of Hsp26 to inhibit the assembly of BMB-crosslinked NM (Figure 3F) suggested that Hsp26 might inhibit fibrillization of NM seeded by preformed NM fibers. Indeed, Hsp26 potently inhibited (IC_50_∼1.9 µM) NM fibrillization seeded by preformed NM fibers (5% wt/wt) (Figure 6A,B). Hsp42 also inhibited seeded assembly (Figure 6A,B). However, Hsp42 was less effective (IC_50_>24 µM) and inhibition was only observed at high concentrations (Figure 6A,B). In contrast to spontaneous assembly (Figure 2A,B), the combination of Hsp26 and Hsp42 did not yield stronger inhibition (Figure 6A,B). Thus, Hsp26 and Hsp42 did not synergize to prevent seeded assembly in vitro (Figure 6A,B), which is consistent with results obtained with BMB-crosslinked NM (Figure 3F). Very similar results were obtained using full-length Sup35 (Figure 6C).

### Thermal Activation of Hsp26 Reduces Activity against Sup35

The strong inhibition of Sup35 prion formation by Hsp26 at 25°C (Figures 2A, 6A) was unexpected because exposure to elevated temperature is required for Hsp26 to bind unfolded polypeptides and prevent their aggregation [27],[31],[32]. Indeed, as expected, pretreatment of Hsp26 for 10 min at 45°C increased the ability of Hsp26 to suppress aggregation of glutamate dehydrogenase (GDH) (Figure 6D). By contrast, the same pretreatment reduced the ability of Hsp26 to inhibit spontaneous NM assembly (Figure 6D). Thus, at elevated temperatures Hsp26 might switch from inhibiting prion assembly to suppressing aggregation of denatured substrates. As with spontaneous NM assembly (Figure 6D), pretreatment of Hsp26 at 45°C reduced its ability (IC_50_>24 µM) to inhibit seeded fibrillization (Figure 6A–C). Thus, conditions that enable Hsp26 to prevent the aggregation of environmentally denatured proteins reduce the ability of Hsp26 to antagonize Sup35 prionogenesis. These data suggest that Hsp26 uses a distinct mechanism to antagonize prion formation.

### Hsp26 Prevents Intermolecular Prion Contact Formation

Prion recognition elements termed the “Head” (residues ∼21–38) and “Tail” (residues ∼91–106) in NM fibers formed at 25°C make homotypic intermolecular contacts such that fibers are constructed by “Head-to-Head” and “Tail-to-Tail” contacts (Figure 1) [40],[41]. We asked whether Hsp26 might inhibit either “Head-to-Head” or “Tail-to-Tail” intermolecular contact formation or both to inhibit seeded fibrillization (Figure 1, step 5). Thus, we employed six different individual NM single cysteine mutants labeled with pyrene in either the head (G25C, G31C, or Q38C) or the tail region (G86C, G96C, or Y106C). Upon intermolecular contact formation and fibrillization, pyrene molecules form excimers (excited-state dimers) that produce a strong red shift in fluorescence [40],[41]. Hsp42 only partially inhibited seeded “Head-to-Head” or “Tail-to-Tail” contact formation (Figure 6E). By contrast, Hsp26 strongly inhibited seeded “Head-to-Head” or “Tail-to-Tail” contact formation, and a 45°C pretreatment reduced this inhibitory activity (Figure 6E).

### Hsp26 Interacts with NM Fibers to Prevent Seeding

**Figure 7:**
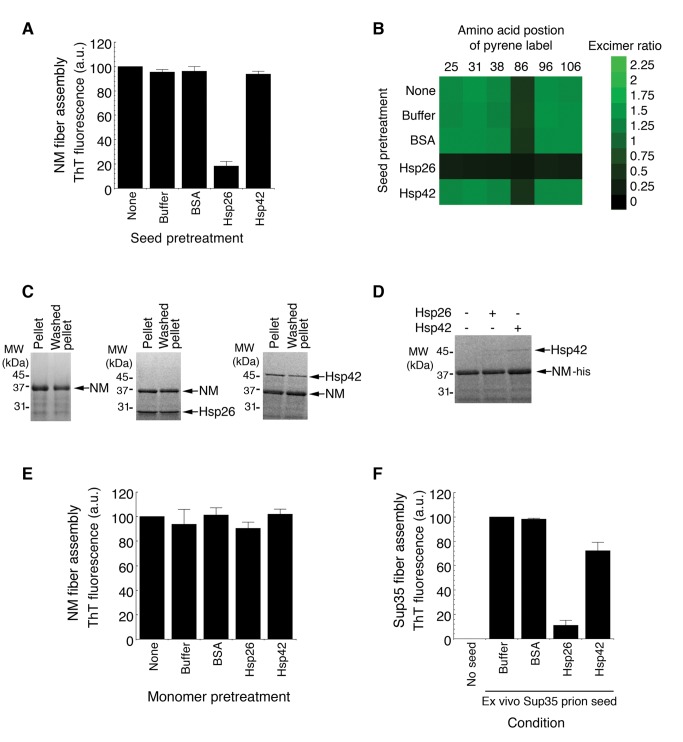
Hsp26 interacts with NM fibers to prevent seeding. (A) NM fibers (5 µM NM monomer) were incubated for 60 min at 25°C without or with BSA, Hsp26, or Hsp42 (10 µM). NM fibers were then recovered by centrifugation at 16,000 g, gently washed, resuspended in an equal volume of assembly buffer, and used to seed (2% wt/wt) assembly of NM (5 µM) for 12 h at 25°C. Fibrillization was measured by ThT fluorescence. Values represent means±SD (*n* = 3). (B) NM fibers were pretreated as in (A) and used to seed (2% wt/wt) assembly of NM proteins (5 µM) carrying pyrene labels at the indicated single site. The ratio of excimer to non-excimer fluorescence (I_465 nm_/I_375 nm_) was then determined as a measure of intermolecular contact formation. (C) Preassembled NM fibers (5 µM NM monomer) were incubated for 60 min at 25°C without or with Hsp26 or Hsp42 (10 µM). NM fibers were then recovered by centrifugation at 16,000 g for 30 min. Pellet fractions with or without a wash step were processed for SDS-PAGE and Coomassie stained. Note the presence of Hsp26 and Hsp42 in the pellet and washed pellet fractions. (D) NM-his (5 µM) was incubated for 60 min at 25°C without or with Hsp26 or Hsp42 (10 µM). NM-his was recovered with Ni-NTA agarose, washed, and eluted. Eluates were processed for SDS-PAGE and Coomassie stained. Note the presence of Hsp42 but not Hsp26 in the eluted fractions. (E) NM-his (5 µM) that had been pretreated with buffer, BSA, Hsp26, or Hsp42 (10 µM) was incubated with NM fibers (5% wt/wt) for 12 h. Fibrillization was measured by ThT fluorescence. Values represent means±SD (*n* = 3). (F) Ex vivo Sup35 prions (2% wt/wt) were used to seed the assembly of soluble full-length Sup35 (5 µM) for 12 h at 25°C in the absence or presence of BSA, Hsp26, or Hsp42 (10 µM). Fibrillization was measured by ThT fluorescence. Values represent means±SD (*n* = 3).

To determine whether Hsp26 inhibited seeded assembly by interacting with NM fibers or monomers or both, we pretreated NM fibers with Hsp26 or Hsp42. We then recovered the fibers by centrifugation, washed, and resuspended the material to use as seed. Hsp26 or Hsp42 did not disassemble NM fibers in this timeframe and equal amounts of NM were recovered for each condition. NM fibers pretreated in this way with BSA or Hsp42 could still seed, whereas those pretreated with Hsp26 were unable to seed (Figure 7A). Very similar results were obtained using pyrene-labeled NM (Figure 7B). Both Hsp42 and Hsp26 were recovered in the pellet with NM fibers (Figure 7C) and thus were present in the seeded assembly reaction. The residual concentration was estimated to be ∼50 nM for Hsp26 and ∼20 nM for Hsp42. These concentrations are not sufficient to cause significant inhibition of seeded assembly without pretreatment of fibers (Figure 6A). Thus, Hsp42 binds to NM fibers in a manner that does not affect seeded assembly. By contrast, Hsp26 binds to NM fibers and occludes prion recognition elements to inhibit seeded assembly.

These results do not exclude that Hsp26 or Hsp42 might also interact with monomeric NM to inhibit prion formation. Thus, we pretreated soluble NM-his with Hsp26 or Hsp42 (or BSA). We then recovered the NM-his using Ni-NTA superflow and used it as substrate for preformed NM fibers. Hsp26 did not bind NM-his monomers (Figure 7D), in contrast to the interaction between Hsp26 and NM fibers (Figure 7C). We detected minimal binding of Hsp42 to NM-his (Figure 7D), but again this interaction was not as pronounced as the interaction between Hsp42 and NM fibers (Figure 7C). The NM-his recovered under each of these conditions was readily converted to amyloid by NM fibers (Figure 7E). These data indicate that the direct interaction between Hsp26 and NM fibers is critical for the inhibition of seeded assembly.

### Hsp26 Prevents Seeding by Ex Vivo Sup35 Prions

Next, we tested whether the sHsps could inhibit seeding by ex vivo Sup35 prions. Several proteins bind Sup35 prions in situ, most notably Ssa1 [75], which might affect how Hsp42 or Hsp26 influence seeding. Hence, we isolated Sup35 prions from [*PSI^+^*] cells [75] and used them to seed the assembly of full-length Sup35 in the absence or presence of Hsp26 or Hsp42. Ex vivo Sup35 prions effectively seeded Sup35 fibrillization and Hsp26 potently inhibited this process (Figure 7F). Hsp42 also inhibited seeding by ex vivo Sup35 prions (Figure 7F) but only at high Hsp42 concentrations and was comparable to the inhibition observed with NM (Figure 6A).

### Elevated Expression of Hsp26 and Hsp42 Effectively Cures [*PSI* ^+^]

**Figure 8:**
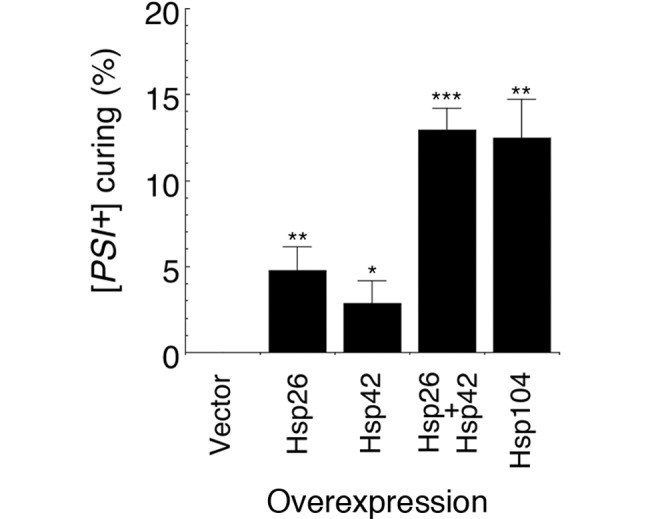
Elevated expression of Hsp26 and Hsp42 effectively cures [*PSI* ^+^]. Hsp26, Hsp42, Hsp26 plus Hsp42, or Hsp104 were overexpressed in [*PSI^+^*] cells in liquid for 6 h at 30°C. Empty vector served as the control. Cells were plated on 25% YPD and the proportion of [*PSI^+^*] colonies was determined. Compared to the vector control there was significantly more [*PSI^+^*] curing in cells expressing Hsp26 (**p = 0.00249, two-tailed Student's *t* test), Hsp42 (*p = 0.0387, two-tailed Student's *t* test), Hsp26 and Hsp42 (***p = 0.000187, two-tailed Student's *t* test), or Hsp104 (**p = 0.00148, two-tailed Student's *t* test). Values represent means±SD (*n* = 3).

The inhibition of seeded assembly by Hsp26 (Figures 6A–C,E, 7F) suggested that Hsp26 might interfere with [*PSI^+^*] propagation in vivo. Indeed, elevated expression of Hsp26 effectively cured [*PSI^+^*], whereas [*PSI^+^*] curing was not detected in the vector control (Figure 8). Surprisingly, even though Hsp42 did not effectively inhibit seeded assembly (Figures 6A–C,E, 7F), elevated expression of Hsp42 also effectively cured [*PSI^+^*] (Figure 8). Moreover, elevated expression of both Hsp26 and Hsp42 cured [*PSI^+^*] just as effectively as overexpression of Hsp104 (Figure 8), which is a potent method of [*PSI^+^*] curing [76]. Thus, Hsp26 and Hsp42 work together to eliminate Sup35 prions in vivo.

### Hsp42 Synergizes with Hsp70 to Antagonize Sup35 Prionogenesis

**Figure 9:**
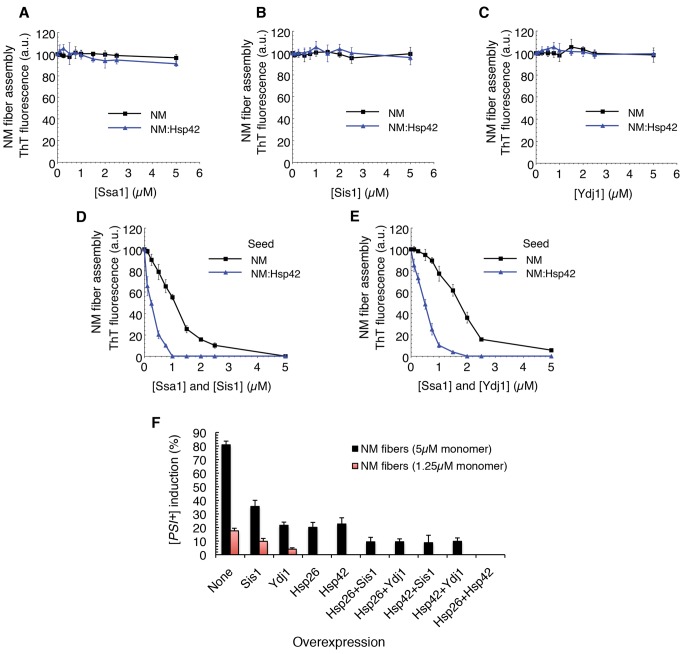
Hsp42 synergizes with Ssa1:Sis1 and Ssa1:Ydj1 to inhibit seeded assembly of NM. (A–E) NM fibers (5 µM NM monomer) were incubated for 60 min at 25°C with buffer or Hsp42 (10 µM). NM fibers were then recovered by centrifugation at 16,000 g, gently washed, resuspended in an equal volume of assembly buffer, and used to seed (2% wt/wt) assembly of NM (5 µM) for 12 h at 25°C in the presence of ATP (5 mM) and increasing concentrations of Ssa1 (A), Sis1 (B), Ydj1 (C), Ssa1:Sis1 (D), or Ssa1:Ydj1 (E). Fibrillization was monitored by ThT fluorescence. Values represent means±SD (*n* = 3). (F) NM fibers (5 µM or 1.25 µM) were sonicated and transformed into [*psi* ^−^] cells overexpressing the indicated combination of Hsp26, Hsp42, Sis1, and Ydj1. The proportion of [*PSI* ^+^] colonies was then determined. Values represent means±SD (*n* = 5).

The magnitude of the [*PSI^+^*] curing effect by Hsp42 overexpression (Figure 8) was unexpected because it is much less effective in inhibiting seeded assembly than Hsp26 (Figures 6A–C,E, 7F). We reasoned that Hsp42 might collaborate with other chaperones to antagonize seeded Sup35 assembly. Ssa1, an Hsp70 chaperone, can collaborate with Hsp40 partners (Sis1 or Ydj1) to inhibit seeded fibrillization of NM [55]. This inhibition is due to an interaction between the fiber and Hsp70 and Hsp40 because neither Ssa1:Ydj1 nor Ssa1:Sis1 interact with NM monomers directly [55]. We tested whether the seeding activity of NM fibers that had been pretreated with Hsp42 were more sensitive to inhibition by increasing concentrations of Ssa1:Sis1 or Ssa1:Ydj1. We first confirmed that adding Ssa1, Ydj1, or Sis1 alone had no effect on seeding by NM fibers or seeding by NM fibers pretreated with Hsp42 (Figure 9A–C). Next, we titrated Ssa1:Sis1 or Ssa1:Ydj1 into seeded assembly while maintaining the Ssa1∶Hsp40 ratio at 1∶1. As expected, both Ssa1:Sis1 and Ssa1:Ydj1 inhibited seeded assembly of buffer-treated NM fibers with an IC_50_ of ∼1.1 µM and ∼1.6 µM, respectively (Figure 9D,E). However, Hsp42-treated NM fibers were more susceptible to inhibition by Ssa1:Sis1 and Ssa1:Ydj1 (Figure 9D,E). The IC_50_ was reduced about 5-fold to ∼0.21 µM for Ssa1:Sis1 and about 3-fold to ∼0.49 µM for Ssa1:Ydj1. These marked decreases in IC_50_ suggest that Hsp42 binds NM fibers and promotes interactions between the prion and Ssa1:Sis1 or Ssa1:Ydj1 that preclude seeded assembly. Thus, Hsp42 may direct Hsp70 and Hsp40 to fiber ends to prevent assembly.

These results were corroborated in vivo. Two titers of synthetic NM prions were transformed into [*psi* ^−^] cells overexpressing the indicated combination of Hsp26, Hsp42, Ydj1, or Sis1 (Figure 9F). Importantly, [*PSI^+^*] induction by NM fibers was reduced by the overexpression of either sHsp or either Hsp40 especially at lower prion titers (Figure 9F). The greatest inhibition was observed when Hsp26 and Hsp42 were combined (Figure 9F). At higher prion titers, the combination of sHsp and Hsp40 was more potent than the sHsp alone (Figure 9F). Specifically, the combination of Hsp26 plus Ydj1 was more effective in preventing infection than Hsp26 alone (two-tailed Student's *t* test; *p* = 0.005), as was Hsp26 plus Sis1 (two tailed Student's *t* test; *p* = 0.0012). Similarly, the combination of Hsp42 plus Ydj1 was more effective in preventing infection than Hsp42 alone (two-tailed Student's *t* test; *p* = 0.006), as was Hsp42 plus Sis1 (two-tailed Student's *t* test; *p* = 0.0025). These data suggest that Hsp42 can collaborate with Hsp70 and Hsp40 to prevent seeding by NM prions in vivo. They also suggest that Hsp26 can prevent seeding by preformed NM prions in vivo.

### Hsp26 and Hsp42 Binding Destabilizes NM Fibers

**Figure 10:**
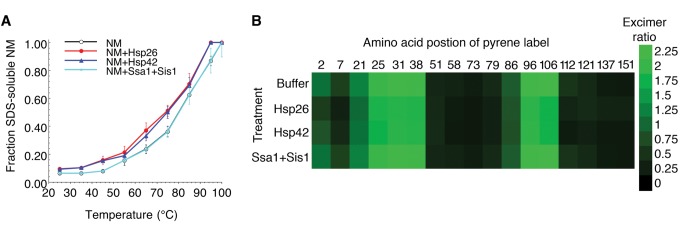
Hsp26 or Hsp42 binding destabilizes NM fibers. (A) NM fibers (5 µM NM monomer) were incubated for 60 min at 25°C without or with Hsp26 (10 µM), Hsp42 (10 µM), or Ssa1:Sis1 (10 µM of each) in the presence of ATP (5 mM). The stability of the various NM fibers was then determined by SDS–PAGE and quantitative immunoblot. The amount of SDS-soluble NM, which reflects susceptibility of NM fibers to thermal solubilization, was plotted against temperature and fitted to a sigmoidal function. Values represent means±SD (*n* = 3). (B) NM proteins (5 µM) carrying pyrene labels at the indicated single site were assembled at 25°C with agitation for 12 h. Assembled NM fibers (5 µM NM monomer) were then incubated for 60 min at 25°C without or with buffer, Hsp26, Hsp42, or Ssa1:Sis1 (10 µM) in the presence of ATP (5 mM). The ratio of excimer to non-excimer fluorescence (I_465 nm_/I_375 nm_) was then determined.

Does Hsp26 or Hsp42 binding alter prion structure? Although Hsp26 and Hsp42 did not disassemble NM fibers after a brief incubation, they both induced a slight decrease in the thermal stability of NM fibers in 1.6% SDS. The melting temperature was slightly reduced from 78±1°C to 71±1°C for Hsp26 and to 72±1°C for Hsp42 (Figure 10A), whereas Ssa1 plus Sis1 had no effect (Figure 10A). Thus, sHsp binding to NM fibers might weaken or subtly alter the intermolecular contacts between NM protomers, and shift the monomer-fiber equilibrium in favor of dissociation. In this way, sHsps might render amyloid forms more susceptible to dissolution by protein disaggregases.

To monitor intermolecular contacts directly, we independently assembled 17 individual single cysteine NM mutants labeled with pyrene [40],[41]. We then determined how Hsp26 and Hsp42 binding affected excimer fluorescence at these positions. Excimer fluorescence detects intermolecular contact integrity and the proximity of residues within different protomers of the assembled prion [40],[41]. Ssa1 and Sis1 had no effect on excimer fluorescence (Figure 10B), whereas Hsp26 and Hsp42 caused subtle but significant alterations in excimer fluorescence at almost every position tested (Figure 10B). Excimer fluorescence in the Head (amino acids 21–38) and Tail (amino acids 86–106) regions was slightly reduced (Figure 10B). The most drastic alteration was observed in the extreme N-terminal positions 2 and 7, where excimer fluorescence was reduced ∼2-fold (Figure 10B). Thus, Hsp26 or Hsp42 binding alters prion architecture in a way that weakens intermolecular contacts and forces residues that are N-terminal to the Head contact (amino acids 21–38) further apart. This sHsp-induced weakening of prion architecture may promote dissolution by prion disaggregases such as Hsp104.

### Hsp26 and Hsp42 Promote Rapid Disassembly of Sup35 Prions by Hsp104

**Figure 11:**
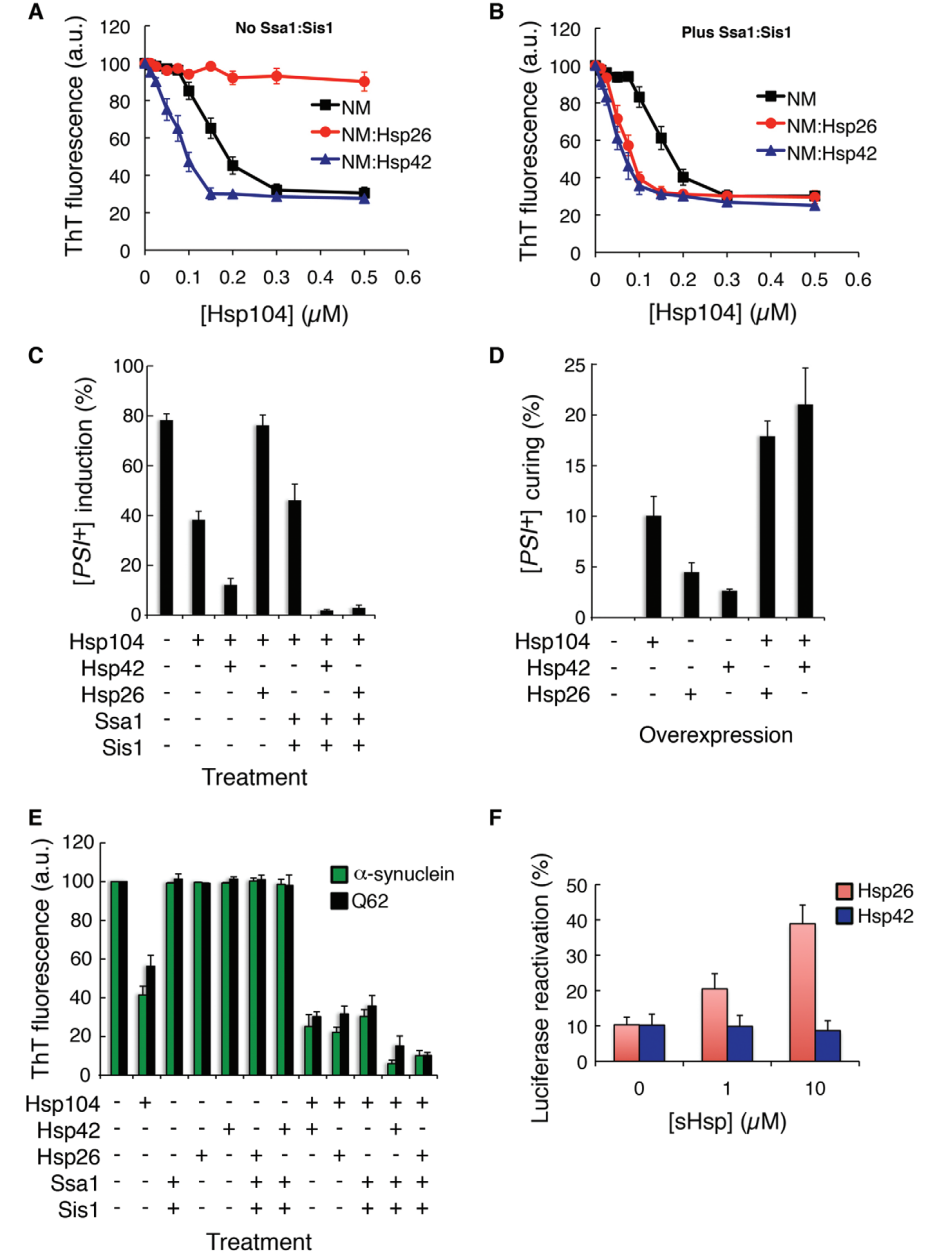
Hsp26 or Hsp42 promotes rapid amyloid disassembly by Hsp104. (A, B) NM fibers (2.5 µM monomer) were pretreated for 1 h at 25°C with either buffer, Hsp26 (10 µM), or Hsp42 (10 µM). Hsp104 (0–0.5 µM) was then added in the absence (A) or presence (B) of Ssa1:Sis1 (2.5 µM each) and reactions were incubated for a further 60 min at 25°C. Fiber integrity was then assessed by ThT fluorescence. Values represent means±SD (*n* = 3). (C) NM fibers (2.5 µM monomer) were pretreated for 1 h at 25°C with either buffer, Hsp26 (10 µM), or Hsp42 (10 µM). Hsp104 (0.15 µM) was then added in the absence or presence of Ssa1:Sis1 (2.5 µM each) and reactions were incubated for a further 60 min at 25°C. Reaction products were concentrated, sonicated, and transformed into [*psi* ^−^] cells. The proportion of [*PSI* ^+^] colonies was then determined. Values represent means±SD (*n* = 3). (D) Hsp104, Hsp26, Hsp42, or the indicated combination were overexpressed in [*PSI^+^*] cells in liquid for 6 h at 30°C. Cells were plated on 25% YPD and the proportion of [*psi* ^−^] colonies was determined. Values represent means±SD (*n* = 3). (E) α-Syn fibers (0.5 µM monomer, green bars) or Q62 fibers (1 µM monomer, black bars) were pretreated for 1 h at 25°C with either buffer, Hsp26 (10 µM), or Hsp42 (10 µM). Hsp104 (10 µM) was then added in the absence or presence of Ssa1:Sis1 (10 µM each) and reactions were incubated for a further 60 min at 25°C (for Q62) or 37°C (for α-syn). Fiber integrity was then assessed by ThT fluorescence. Values represent means±SD (*n* = 3). (F) Luciferase (0.1 µM) was aggregated for 15 min at 45°C in the presence of the indicated concentration of sHsp (Hsp26 or Hsp42). Protein aggregates were diluted 20-fold into chaperone mixtures containing Hsp104 (1 µM), Ssa1 (1 µM), and Ydj1 (1 µM) plus ATP (5 mM) and incubated for 90 min at 25°C. Native luciferase activity at the same concentration was set to 100%. Note that Hsp26 stimulates luciferase reactivation, whereas Hsp42 does not. Note that Hsp104 alone or Ssa1 and Ydj1 alone do not promote luciferase reactivation under these conditions [33].

The destabilization of Sup35 prions by Hsp26 or Hsp42 binding could be exploited by Hsp104 to rapidly disaggregate prions. Thus, we assembled NM fibers and incubated them with increasing concentrations of Hsp104 in the presence or absence of Hsp26 or Hsp42. In the absence of other components Hsp104 effectively disassembled NM fibers, with an EC_50_ of ∼0.15 µM (Figure 11A). Remarkably, Hsp42 enabled Hsp104 to disassemble NM fibers at concentrations where it would usually be inactive (0.01–0.1 µM; Figure 11A). Hsp42 reduced the Hsp104 EC_50_ to ∼0.075 µM. By contrast, Hsp26 inhibited Hsp104 activity (Figure 11A). Addition of Ssa1:Sis1 had little effect on Hsp104 activity in the absence of sHsps (the Hsp104 EC_50_ was ∼0.14 µM; Figure 11B). However, Ssa1:Sis1 enhanced Hsp104 activity in the presence of Hsp42 and reduced the Hsp104 EC_50_ to ∼0.05 µM (Figure 11B). Ssa1:Sis1 relieved the inhibition of Hsp104 by Hsp26 and potentiated Hsp104 remodeling activity, reducing the EC_50_ to ∼0.06 µM. We confirmed that prions had been protected or eliminated by transforming products into [*psi* ^−^] cells (Figure 11C). These data suggest that sHsps enhance the ability of Hsp104 to eliminate Sup35 prions.

Next, we tested whether overexpression of Hsp26 or Hsp42 increased [*PSI^+^*] curing by elevated Hsp104 concentrations. Consistent with our in vitro observations, Hsp26 and Hsp42 synergized with Hsp104 to promote [*PSI^+^*] curing (Figure 11D). These data reinforce our in vitro observations that sHsps potentiate Hsp104 activity against Sup35 prions.

### Hsp26 and Hsp42 Promote Rapid Disassembly of Diverse Amyloids by Hsp104

Next, we tested whether the sHsps potentiated Hsp104 activity against α-syn amyloid, which is connected to Parkinson's disease [1],[77],[78], and polyglutamine amyloid, which is connected to Huntington's disease [1],[79]. α-Syn and polyglutamine (Q62) fibers were assembled and preincubated with either Hsp26 or Hsp42. The indicated combination of Hsp104, Ssa1, and Sis1 was then added. sHsps promoted rapid disassembly of α-syn and polyglutamine fibers by Hsp104 (Figure 11E). Indeed, preincubation with Hsp26 enabled Hsp104 to catalyze more α-syn fiber disassembly (two-tailed Student's *t* test; *p* = 0.0031) and more Q62 fiber disassembly (two-tailed Student's *t* test; *p* = 0.0037) than Hsp104 alone. For these amyloid substrates, Hsp26 alone did not antagonize Hsp104 activity (Figure 11E). Preincubation of fibers with Hsp42 also enabled Hsp104 to catalyze more α-syn fiber disassembly (two-tailed Student's *t* test; *p* = 0.0209) and more Q62 fiber disassembly (two-tailed Student's *t* test; *p* = 0.0019) than Hsp104 alone. Optimal disaggregation was achieved with sHsp plus Hsp104, Ssa1, and Sis1 (Figure 11E). Thus, sHsps potentiate Hsp104 activity against disease-associated amyloid.

### Hsp42 Is an Amyloid-Specific Adaptor for Hsp104

Next, we asked whether Hsp42 could function like Hsp26 to promote the disaggregation of disordered aggregates by Hsp104 [33]. In contrast to Hsp26, Hsp42 did not promote the disaggregation of heat-denatured luciferase aggregates by Hsp104, Hsp70, and Hsp40 (Figure 11F). These data help explain why Hsp26, but not Hsp42, assists Hsp104 in promoting luciferase disaggregation and thermotolerance in vivo [33]. Thus, Hsp42 selectively promotes the disassembly of amyloid conformers by Hsp104, whereas Hsp26 promotes Hsp104-catalyzed disaggregation of both amyloid and non-amyloid aggregates.

### Hsp110, Hsp70, and Hsp40 Promote Slow Disassembly of NM Fibers

**Figure 12:**
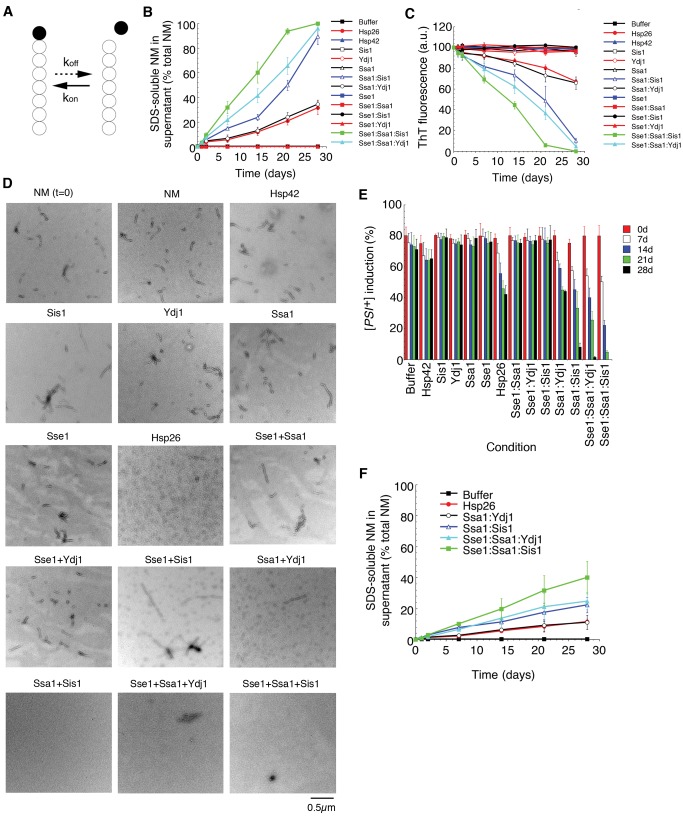
Gradual disassembly of Sup35 prions by Hsp26 or Hsp110, Hsp70, and Hsp40. (A) Molecular recycling model. Monomers at the fiber ends are constantly dissociating and reassociating. An example monomer is shown in black. *k*_off_ is the rate constant for the dissociation of a monomer from a fiber end and *k*_on_ is the rate constant for the association of a monomer with a fiber end. *k*_on_ is several orders of magnitude greater than *k*_off_. (B–E) NM fibers (2.5 µM) were sonicated and then incubated at 25°C for 0–28 d in the absence or presence of Hsp26 (5 µM), Hsp42 (5 µM), Sis1 (5 µM), Ydj1 (5 µM), Ssa1 (5 µM), Sse1 (5 µM), Sse1:Ssa1, Sse1:Sis1, Sse1:Ydj1, Ssa1:Sis1 (2.5 µM each), Ssa1:Ydj1 (2.5 µM each), Sse1:Ssa1:Sis1 (1.67 µM each), or Sse1:Ssa1:Ydj1 (1.67 µM each). At the indicated times, reactions were centrifuged at 436,000 g for 30 min. The amount of SDS-soluble NM in the supernatant was then determined by quantitative immunoblot (B). Values represent means±SD (*n* = 3). Alternatively, at the indicated times, fiber integrity was assessed by ThT fluorescence (C). Values represent means±SD (*n* = 3). After 28 d, fiber integrity was assessed by electron microscopy (D). Sonicated NM fibers at the start of the reaction (t = 0) are shown at the top left for comparison. Note the absence of fibers in the presence of Sse1:Ssa1:Sis1, Sse1:Ssa1:Ydj1, and Ssa1:Sis1 and a collection of shorter forms in the presence of Hsp26 or Ssa1:Ydj1. Bar, 0.5 µm (D). Alternatively, at the indicated times, reaction products were concentrated and transformed into [*psi* ^−^] cells. The proportion of [*PSI* ^+^] colonies was then determined (E). Values represent means±SD (*n* = 3). (F) NM fibers (2.5 µM) were *not* sonicated and then incubated at 25°C for 0–28 d in the absence or presence of Hsp26 (5 µM), Ssa1:Sis1 (2.5 µM each), Ssa1:Ydj1 (2.5 µM each), Sse1:Ssa1:Ydj1 (1.67 µM each), or Sse1:Ssa1:Sis1 (1.67 µM each). At the indicated times, reactions were centrifuged at 436,000 g for 30 min. The amount of SDS-soluble NM in the supernatant was then determined by quantitative immunoblot. Values represent means±SD (*n* = 3). Note the extent of disassembly is not as extensive as when NM fibers are sonicated as in (B).

Metazoa lack an Hsp104 orthologue and how amyloid might be disaggregated in animal systems remains unknown [60]. In general, monomers at fiber ends are more likely to be susceptible to disaggregation because they are only restrained by one intermolecular contact (e.g., Head or Tail, Figure 1). Indeed, fiber ends are dynamic and monomers slowly dissociate within a biologically relevant timeframe (days) and rapidly reassociate in a process termed molecular recycling (Figure 12A) [62]–[64],[67],[68]. Dissociation is the rate-limiting step in recycling and reassociation is rapid. A homogeneous population of fibers formed by a SH3 domain with an average length of 100 nm, recycle ∼50% of monomers within 2 to 20 d [62]. Thus, agents that accelerate monomer dissociation or prevent monomer reassociation or both could drive fiber depolymerization on a timescale similar to that of molecular recycling. For example, Hsp26 and Hsp70:Hsp40 pairs (e.g., Ssa1:Sis1) prevent seeded assembly (Figures 6A–C, 9D, 9E) and might inhibit monomer reassociation events (Figure 12A). We were particularly interested in Hsp110 in this context. Hsp110 can synergize with Hsp70 and Hsp40 to extract and refold proteins from denatured aggregates [61]. Thus, the combination of Hsp110, Hsp70, and Hsp40 might even accelerate dissociation of monomers from fiber ends.

We assembled and sonicated NM fibers to generate a uniform population of short fibers [43]. NM fibers were stable for 28 d alone (black filled squares, Figure 12B,C) or in the presence of molecular chaperones that alone do not affect seeded assembly, including Hsp42 (blue filled triangles, Figure 12B,C), Sis1 (black open squares, Figure 12B,C), Ydj1 (red open circles, Figure 12B,C), Ssa1 (black open triangles, Figure 12B,C), or Sse1 (blue filled squares, Figure 12B,C). In remarkable contrast, Hsp26 (red filled circles, Figure 12B,C), Ssa1:Sis1 (blue open triangles, Figure 12B,C), and Ssa1:Ydj1 (black open circles, Figure 12B), which inhibit seeded assembly (6A, 6B, 9D, 9E), slowly disassembled preformed NM fibers over a time period of 28 d (Figure 12B,C). Consistent with their efficacy to inhibit seeded assembly, Ssa1:Sis1 (blue open triangles, Figure 12B,C) was more effective than Ssa1:Ydj1 (black open circles, Figure 12B,C) or Hsp26 (red filled circles, Figure 12B,C). Notably, the combination of Sse1 (Hsp110), Ssa1 and Sis1 (green filled squares, Figure 12B,C), or Sse1, Ssa1, and Ydj1 (cyan filled triangles, Figure 12B,C) yielded more rapid disassembly, whereas Sse1 combined with Ssa1 (red filled squares, Figure 12B,C), Sis1 (black filled circles, Figure 12B,C), or Ydj1 (red filled triangles, Figure 12B,C) had no effect. Electron microscopy (Figure 12D) and prion transformation (Figure 12E) confirmed that prions had been eliminated by Sse1:Ssa1:Sis1, Sse1:Ssa1:Ydj1, Ssa1:Sis1, Ssa1:Ydj1, and Hsp26, but not by Sse1:Ssa1, Sse1:Sis1, Sse1:Ydj1, Sse1, Ssa1, Ydj1, Sis1, or Hsp42.

**Figure 13 pbio-1001346-g013:**
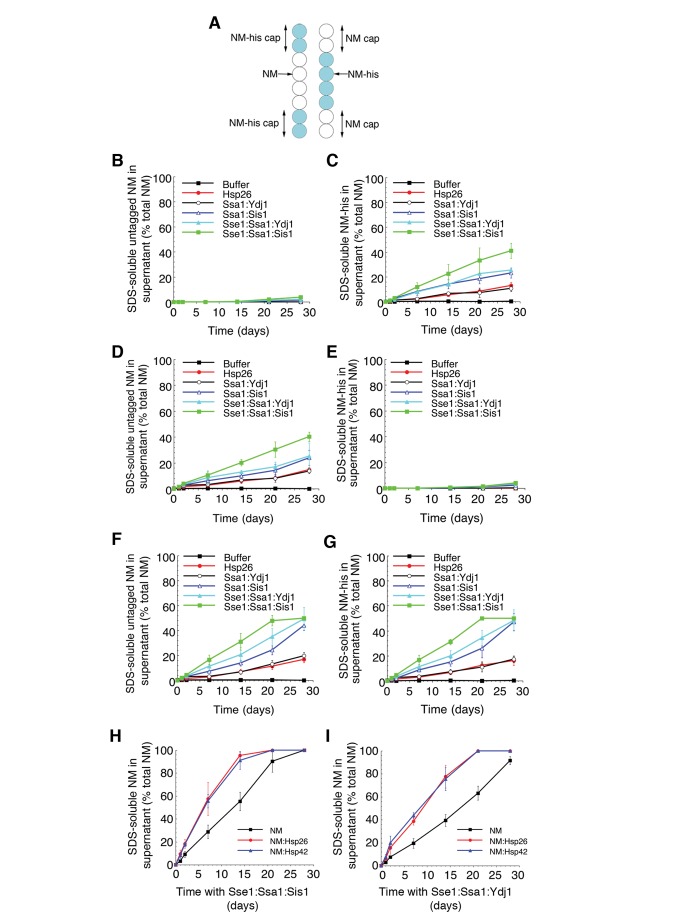
sHsps promote gradual depolymerization of Sup35 prions by Hsp110, Hsp70, and Hsp40. (A) Schematic illustrating the concept of NM-his capped NM fibers (left) or NM capped NM-his fibers (right). Cyan circles depict NM-his and white circles depict NM. (B, C) NM fibers with NM-his caps (2.5 µM monomer) were *not* sonicated and incubated at 25°C for 0–28 d in the absence or presence of Hsp26 (5 µM), Ssa1:Sis1 (2.5 µM each), Ssa1:Ydj1 (2.5 µM each), Sse1:Ssa1:Ydj1 (1.67 µM each), or Sse1:Ssa1:Sis1 (1.67 µM each). At the indicated times, reactions were centrifuged at 436,000 g for 30 min. The amount of SDS-soluble untagged NM (B) or NM-his (C) in the supernatant was then determined by quantitative immunoblot. Values represent means±SD (*n* = 3). (D, E) NM-his fibers with NM caps (2.5 µM monomer) were *not* sonicated and incubated at 25°C for 0–28 d in the absence or presence of Hsp26 (5 µM), Ssa1:Sis1 (2.5 µM each), Ssa1:Ydj1 (2.5 µM each), Sse1:Ssa1:Ydj1 (1.67 µM each), or Sse1:Ssa1:Sis1 (1.67 µM each). At the indicated times, reactions were centrifuged at 436,000 g for 30 min. The amount of SDS-soluble untagged NM (D) or NM-his (E) in the supernatant was then determined by quantitative immunoblot. Values represent means±SD (*n* = 3). (F, G) NM-his fibers with NM caps (2.5 µM monomer) *were* sonicated and incubated at 25°C for 0–28 d in the absence or presence of Hsp26 (5 µM), Ssa1:Sis1 (2.5 µM each), Ssa1:Ydj1 (2.5 µM each), Sse1:Ssa1:Ydj1 (1.67 µM each), or Sse1:Ssa1:Sis1 (1.67 µM each). At the indicated times, reactions were centrifuged at 436,000 g for 30 min. The amount of SDS-soluble untagged NM (F) or NM-his (G) in the supernatant was then determined by quantitative immunoblot. Values represent means±SD (*n* = 3). (H, I) NM fibers (2.5 µM) were sonicated and then incubated for 1 h at 25°C with buffer, Hsp26, or Hsp42 (10 µM). (H) Sse1:Ssa1:Sis1 (1.67 µM each) or (I) Sse1:Ssa1:Ydj1 (1.67 µM each) was then added and fibers were incubated for 0–28 d at 25°C. At the indicated times, reactions were centrifuged at 436,000 g for 30 min. The amount of SDS-soluble NM in the supernatant was then determined by quantitative immunoblot. Values represent means±SD (*n* = 3).

Disassembly was contingent on the number of fiber ends, as unsonicated fibers were more refractory to disassembly (Figure 12F). Indeed, we confirmed that disassembly entailed depolymerization from fiber ends using “capped” fibers. Thus, NM fibers comprised of untagged NM were resuspended in buffer containing high concentrations of C-terminally his-tagged NM. This procedure allowed NM fibers to be rapidly elongated creating NM fibers with NM-his “caps” (Figure 13A). We established conditions where ∼50% of the total NM in fibers was his-tagged (Figure 13A). If NM fibers with NM-his “caps” were treated for extended periods with Sse1:Ssa1:Sis1, Sse1:Ssa1:Ydj1, Ssa1:Sis1, Ssa1:Ydj1, or Hsp26, then only NM-his was released into the soluble fraction (Figure 13B,C). Conversely, if NM-his fibers were capped with untagged NM (Figure 13A), then only untagged NM was released into the soluble fraction (Figure 13D,E). When capped fibers were sonicated prior to incubation to randomize the form of NM at fiber ends, approximately equal amounts of NM and NM-his were released (Figure 13F,G). Taken together, these data suggest that Sse1:Ssa1:Sis1, Sse1:Ssa1:Ydj1, Ssa1:Sis1, Ssa1:Ydj1, and Hsp26 slowly depolymerize NM fibers from their ends. The most effective depolymerization is promoted by the combination of Hsp110, Hsp70, and Hsp40.

### sHsps Can Promote Depolymerization of NM Fibers by Hsp110, Hsp70, and Hsp40

Hsp110, Hsp70, and Hsp40 might exploit the destabilization of amyloid by sHsp binding (Figure 10A,B) to promote amyloid depolymerization. Thus, NM fibers were pretreated with Hsp26 or Hsp42 prior to addition of Sse1:Ssa1:Sis1 or Sse1:Ssa1:Ydj1. Pretreatment with Hsp26 or Hsp42, which subtly alters NM fiber structure and stability (Figure 10A,B), facilitated more rapid depolymerization by Sse1:Ssa1:Sis1 (Figure 13H) or Sse1:Ssa1:Ydj1 (Figure 13I). For Sse1:Ssa1:Sis1, the D_1/2_ (the 50% disassembly time) was reduced from ∼12.8 d to ∼7.4 d by Hsp26 and to ∼7.7 d by Hsp42. For Sse1:Ssa1:Ydj1, D_1/2_ was reduced from ∼16.1 d to ∼10.5 d by Hsp26 and ∼10.9 d by Hsp42. Thus, sHsps render amyloid forms more susceptible to depolymerization by the Hsp110, Hsp70, and Hsp40 disaggregase machinery.

### Human HspB5 Promotes Depolymerization of α-Syn Fibers by Human Hsp110, Hsp70, and Hsp40

**Figure 14:**
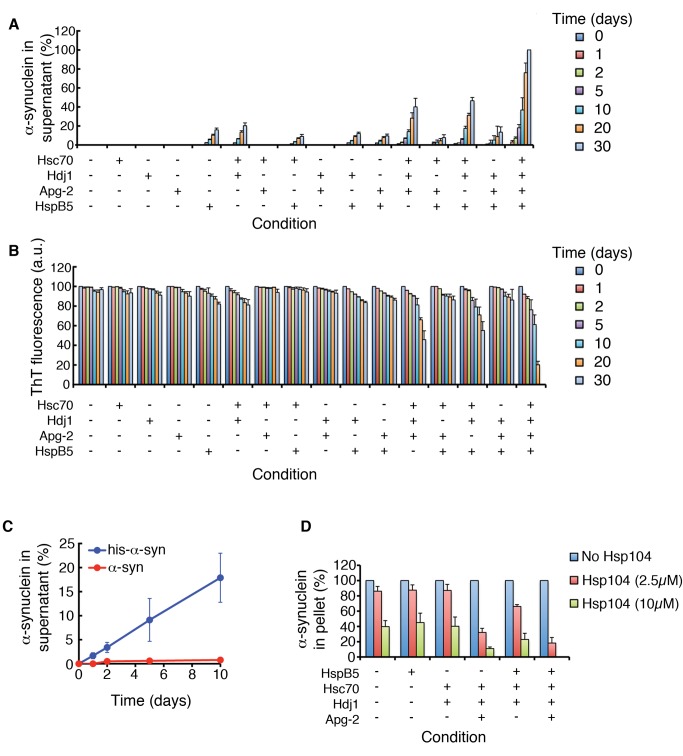
Human HspB5 promotes gradual depolymerization of α-syn fibers by human Hsp110, Hsp70, and Hsp40. (A, B) α-Syn fibers (2.5 µM monomer) were sonicated and then incubated for 0–30 d at 25°C with either buffer, Hsc70 (10 µM), Hdj1 (10 µM), Apg-2 (10 µM), HspB5 (10 µM), Hsc70:Hdj1 (5 µM of each), Hsc70:Apg-2 (5 µM of each), Hsc70:HspB5 (5 µM of each), Hdj1:Apg-2 (5 µM of each), Hdj1:HspB5 (5 µM of each), Apg-2:HspB5 (5 µM of each), Hsc70:Hdj1:Apg-2 (3.3 µM of each), Hsc70:Apg-2:HspB5, Hsc70:Hdj1:HspB5, Hdj1:Apg-2:HspB5 (3.3 µM of each), or Hsc70:Hdj1:Apg-2:HspB5 (2.5 µM of each). At the indicated times, reactions were centrifuged at 436,000 g for 30 min (A). The amount of α-syn in the supernatant was then determined by quantitative immunoblot (A). Alternatively, fiber integrity was assessed by ThT fluorescence (B). Values represent means±SD (*n* = 3). (C) α-Syn fibers with his-α-syn caps (2.5 µM monomer) were *not* sonicated and incubated at 25°C for 0–30 d in the presence of Hsc70:Hdj1:Apg-2:HspB5 (2.5 µM of each). At the indicated times, reactions were centrifuged at 436,000 g for 30 min. The amount of his-α-syn (blue) or untagged α-syn (red) in the supernatant was then determined by quantitative immunoblot. Values represent means±SD (*n* = 3). (D) Preformed amyloid fibers composed of α-syn (0.5 µM monomer) were incubated for 6 h at 37°C in buffer plus ATP (5 mM) and ATP-regeneration system without or with the indicated combination of HspB5 (10 µM), Hsc70 (10 µM), Hdj1 (10 µM), and Apg-2 (10 µM) in the absence (blue bars) or presence of Hsp104 (2.5 µM, red bars; or 10 µM, green bars). Fiber integrity was then determined by sedimentation analysis and quantitative immunoblot. Values represent means±SD (*n* = 3).

Our newly discovered amyloid-depolymerase activity was not restricted to yeast chaperones and yeast prions. Indeed, human Hsp70 (Hsc70) and Hsp40 (Hdj1) slowly disassemble preformed α-syn fibers (Figure 14A,B). This activity was stimulated by addition of human Hsp110 (Apg-2) or the human sHsp, HspB5 (Figure 14A,B). HspB5 potentiated α-syn fiber disassembly by Apg-2, Hsc70 and Hdj1 (Figure 14A,B). Indeed, HspB5 reduced the D_1/2_ to ∼14 d for Apg-2, Hsc70, and Hdj1. We confirmed that the combination of Apg-2, Hsc70, Hdj1, and HspB5 depolymerized fibers from their ends by employing α-syn fibers capped with his-α-syn. Thus, during the initial disassembly phase of unsonicated fibers, Apg-2, Hsc70, Hdj1, and HspB5 liberated only his-α-syn into the soluble fraction (Figure 14C), which indicates that disassembly proceeds via depolymerization. These data suggest that the human proteostasis network, like its yeast counterpart, is equipped with an amyloid-depolymerase modality. Although depolymerization is relatively slow, it occurs on a biologically relevant timescale, especially considering the lifespan of neurons in the human brain.

Could Hsp104 interface with the human sHsp, HspB5, and the Hsp110, Hsp70, and Hsp40 disaggregase machinery? Remarkably, the combination of Apg-2, Hsc70, Hdj1, and HspB5 enabled even more effective and rapid disaggregation of α-syn fibers by Hsp104 than with just Apg-2, Hsc70, and Hdj1 (Figure 14D) [61]. The ability of Hsp104 to interface effectively with the human disaggregase machinery and enable effective clearance of α-syn amyloid suggests that Hsp104 might be developed further to target pathological α-syn conformers. Collectively, our studies suggest that sHsps are ubiquitous potentiators of amyloid disassembly by the proteostasis network.

## Discussion

We have established that the sHsps from yeast, Hsp26 and Hsp42, exert tight control over the formation of beneficial Sup35 prions. Both sHsps exerted a strong and direct inhibitory effect on Sup35 prion formation at substoichiometric concentrations. These results were surprising because sHsps commonly bind 1 substrate per ∼2–3 sHsp monomers [24],[27]. Thus, the strong inhibitory effect at substoichiometric concentrations indicates that the sHsps might inhibit a rare or transient NM conformer that is critical for prion formation. Surprisingly, our results suggested that this conformer was different for each sHsp. Hsp42 targeted molten Sup35 oligomers, whereas Hsp26 targeted the self-templating ends of newly assembled prions (Figure 1).

Although little was known about Hsp42, it had been suggested to work by a mechanism similar to Hsp26 to inhibit protein aggregation [28]. Surprisingly, however, Hsp42 inhibited spontaneous Sup35 prionogenesis by a distinct mechanism to Hsp26. Hsp42 specifically antagonized events in the lag phase of prion formation. Hsp42 prevented and reversed the maturation of Sup35 oligomers into prion-nucleating species (Figure 1, steps 2 and 3). By contrast, Hsp26 bound to newly formed prions and inhibited their seeding activity (Figure 1, step 5). These two activities synergized to inhibit *de novo* Sup35 prionogenesis in vitro and in vivo. To the best of our knowledge, this is the first example of two distinct sHsps working together in a synergistic manner to prevent prion formation.

The mechanistic differences between Hsp26 and Hsp42 are likely conferred by their divergent N-terminal domains. Hsp42 has an extended N-terminal domain [80], which displays no homology to other sHsps. The extended N-terminal domain of Hsp42 might enable insertion into molten Sup35 oligomers in a way that precludes prion formation.

Hsp26 chaperone activity is usually activated at heat shock temperatures [27],[29],[31],[32]. Unexpectedly, we found that pretreatment of Hsp26 at high temperature reduced its ability to inhibit Sup35 prionogenesis, while simultaneously enhancing its ability to prevent aggregation of a chemically denatured substrate. Our result thus reveals a fundamental difference in how Hsp26 antagonizes the aggregation of a denatured protein (GDH) and a yeast prion (Sup35). Hsp26 conformations that are ineffective against heat-denatured substrates are effective against Sup35 prions and vice versa. This difference might reflect distinct driving forces of GDH and Sup35 aggregation. GDH aggregation likely involves inappropriately exposed hydrophobic surfaces, whereas NM fibrillization likely involves polar interactions or backbone interactions or both because polar residues outweigh hydrophobic residues by ∼16 to 1. At physiological temperatures, Hsp26 may be primed to inhibit prion formation, but at elevated temperatures, Hsp26 loses this ability and switches to inhibiting the aggregation of heat-denatured proteins. This switch in Hsp26 activity likely contributes to the increased levels of [*PSI^+^*] induction at elevated temperatures [11].

Both Hsp26 and Hsp42 bind to preformed Sup35 fibers, but only Hsp26 binding inhibited seeding activity. However, Hsp42 increased the ability of Hsp70 and Hsp40 (Ssa1:Sis1 or Ssa1:Ydj1) to inhibit seeded assembly, potentially by recruiting Hsp70 to fiber ends. These data help explain why overexpression of Hsp26 or Hsp42 cures cells of [*PSI^+^*].

Unexpectedly, Hsp26 or Hsp42 binding destabilized Sup35 prions. Hsp26 and Hsp42 binding reduced excimer fluorescence at intermolecular contact regions. The most marked effect was at residues N-terminal to the Head region, which appeared to be forced further apart in adjacent protomers by Hsp26 or Hsp42 binding. These data suggest that Hsp26 or Hsp42 harness binding energy to alter prion architecture. Notably, these sHsp-induced alterations facilitated the disaggregation of Sup35 prions by Hsp104.

Pretreatment of Sup35 prions with Hsp42 rendered them more susceptible to rapid disassembly by Hsp104. Curiously, Hsp26 alone inhibited Hsp104. However, Ssa1 and Sis1 alleviated this inhibition and promoted more effective prion disassembly. These findings might suggest that the mechanism of Sup35 prion disassembly by Hsp104 is different in the presence of Hsp26 versus Hsp42. Further experiments are needed to explore this possibility. Importantly, Hsp26 and Hsp42 promoted elimination of Sup35 prions by Hsp104 in vivo, as overexpression of Hsp26 or Hsp42 increased [*PSI^+^*] curing by elevated Hsp104 concentration.

Hsp26 and Hsp42 also promoted rapid Hsp104-catalyzed disassembly of α-syn fibers that are connected with PD. We further demonstrated that Hsp104 directly disassembles polyglutamine fibers that are connected with HD. Hsp26 or Hsp42 boosted this activity and disaggregation was maximal in the presence of Hsp104, an sHsp, Ssa1, and Sis1.

We have established an important dichotomy between how Hsp26 and Hsp42 collaborate with Hsp104. Hsp26 promotes the disaggregation of both amyloid and non-amyloid substrates by Hsp104 in the presence of Hsp70 and Hsp40. By contrast, Hsp42 selectively promotes the disassembly of amyloid substrates by Hsp104. Thus, Hsp42 is an amyloid-specific adaptor for Hsp104. In yeast, Hsp42 appears to preferentially localize to peripheral inclusions [34] that might harbor amyloid conformers that can be solubilized by Hsp104 [53],[56],[81],[82].

We have shown that in the absence of Hsp104, the Hsp110, Hsp70, and Hsp40 disaggregase system [61] can slowly depolymerize amyloid fibers. Depolymerization was a slow process that required many days to complete and appeared to occur on a timescale similar to molecular recycling within amyloid fibers [62],[67]. Thus, the proteostasis network might exploit this process to slowly eradicate amyloid by either accelerating monomer dissociation from fiber ends (i.e., increasing *k*_off_, Figure 12A) or inhibiting monomer reassociation with fiber ends (i.e., decreasing *k*_on_, Figure 12A) or both. Consistent with the possibility of inhibiting monomer reassociation (decreasing *k*_on_, Figure 12A), agents that inhibit seeded polymerization of Sup35 prions (e.g., Hsp26 or Ssa1:Sis1) slowly depolymerized them over the course of many days. The relatively low number of Hsp26 monomers per molecule of substrate required for Hsp26 disaggregation activity might indicate that Hsp26 acts selectively at fiber ends. The combination of Sse1, Ssa1, and Sis1 yielded the most effective depolymerization. Given the capability of this disaggregase system to extract and refold proteins from large denatured aggregates [61], we suggest that Hsp110, Hsp70, and Hsp40 might also accelerate monomer dissociation events (increasing *k*_off_, Figure 12A). Importantly, destabilization of NM fibers by Hsp26 or Hsp42 accelerated prion depolymerization by Hsp110, Hsp70, and Hsp40.

Intriguingly, this activity is not confined to yeast but is conserved to humans. Thus, the human sHsp, HspB5, accelerated the depolymerization of α-syn amyloid (which is connected with PD) by human Hsp110 (Apg-2), Hsp70 (Hsc70), and Hsp40 (Hdj1). Collectively, these data suggest that in metazoa, which lack an Hsp104 homologue, Hsp110, Hsp70, and Hsp40 can slowly eliminate amyloid forms by specifically exploiting the molecular recycling process (Figure 12A). Although amyloid depolymerization is slow and requires many days to complete, it occurs on a biologically relevant timescale, especially considering the lifespan of humans. Indeed, a massive therapeutic advance will likely come with the ability to stimulate the proteostasis network to dissolve α-syn fibers in a few days in Parkinson's patients. Our data provide proof of principle that this may indeed be possible and that pure, individual components can drive this process. Although released monomers could have a chance to reassemble into toxic oligomers, we suspect that components of the proteostasis network would prevent toxic oligomer formation. Shutting down expression of an amyloidogenic protein enables mammalian cells to slowly clear protein aggregates [83],[84]. Our findings suggest that sHsps and the Hsp110, Hsp70, and Hsp40 disaggregase system might play a crucial role in this clearance. Moreover, they suggest that potential RNA interference therapies to deplete the aggregating protein should be combined with targeted upregulation of sHsps and the Hsp110, Hsp70, and Hsp40 disaggregase system to promote clearance of existing aberrant conformers.

Another way to accelerate the disaggregation of α-syn fibers is to introduce Hsp104 [54],[61]. Indeed, the combination of Hsp104 with Apg-2, Hsc70, Hdj1, and HspB5 disaggregated α-syn fibers most effectively and rapidly. Importantly, Hsp104 expression counteracts neurodegeneration associated with α-syn misfolding and polyglutamine misfolding in rodents [54],[59],[85],[86]. Thus, our findings suggest that boosting sHsp levels or activity might provide a powerful strategy to facilitate clearance of deleterious amyloid by either the endogenous human Hsp110, Hsp70, and Hsp40 disaggregase machinery [61] or by Hsp104 in targeted therapeutic strategies [54],[59],[85]–[87].

## Materials and Methods

### Proteins

Hsp26 [27], Hsp42 [28], Ssa1, Sis1, Ydj1, NM [48], NM-his [51], Sup35 [49], Hsp104 [88], Apg-2 [89], Sse1 [90], Rnq1 [74], polyglutamine (GST-Q62) [91], and α-syn [92] were purified as described. Hsc70 and Hdj1 were from Enzo Life Sciences. HspB5 was from ProSpec. BSA and firefly luciferase were from Sigma and GDH was from Roche. Single cysteine NM mutants were labeled with pyrene-maleimide or acrylodan (Invitrogen) or crosslinked with BMB (Pierce) under denaturing conditions as described [40]. Throughout the manuscript, protein concentrations refer to the monomer, with the exception of Hsp104, where it refers to the hexamer.

### Plasmids for Chaperone Overexpression in Yeast

The plasmids used for overexpression of Hsp26, Hsp42, Hsp104, Ydj1, Sis1, Sup35, and NM-YFP were as described [33],[41],[90],[93].

### GDH Aggregation

The aggregation of denatured GDH was monitored by turbidity at 395 nm [31]. In some experiments, Hsp26 was thermally activated by incubation at 45°C for 10 min prior to addition to aggregation assays.

### Fiber Assembly

NM (5 µM) fibrillization was conducted in assembly buffer (AB) (40 mM HEPES-KOH, pH 7.4, 150 mM KCl, 20 mM MgCl_2_, 1 mM DTT). For Sup35 (5 µM) fibrillization, AB was supplemented with 1 mM GTP and 10% glycerol. Unseeded reactions were agitated at 1,400 r.p.m. (for NM) or 700 r.p.m. (for Sup35) in a thermomixer (Eppendorf) for the indicated time at 25°C. Seeded assembly was unagitated and performed for the indicated time at 25°C. The amount of seed is indicated as % (wt/wt). In some experiments (Figure 7A), NM fibers (5 µM NM monomer) were pretreated for 60 min at 25°C without or with BSA, Hsp26, or Hsp42 (10 µM). NM fibers were then recovered by centrifugation at 16,000 g, gently washed (without resuspending the pellet), and then resuspended in AB. Ex vivo Sup35 prions for seeding experiments were isolated as described [75] and the amount of Sup35 in the isolated fraction was determined by immunoblot in comparison to known quantities of pure Sup35. Rnq1 fibers were assembled as described [74]. For assembly reactions containing Ssa1:Sis1 or Ssa1:Ydj1, ATP was added (5 mM) plus an ATP regeneration system comprising creatine phosphate (40 mM) and creatine kinase (0.5 µM). The extent of fiber assembly was determined by ThT fluorescence, electron microscopy, or by the amount of SDS-resistant NM as described [49],[55].

### Tracking Amyloidogenic NM Oligomers

The oligomer-specific A11 antibody was used to detect amyloidogenic NM oligomers by ELISA as described [69]. Importantly, Hsp26 and Hsp42 did not cross-react with A11.

### Fiber Disassembly

For NM disassembly reactions, NM (5 µM) was assembled with agitation for 6 h in AB as described above. Wild-type or A53T α-syn fibers were assembled as described [54]. Polyglutamine (GST-Q62) (10 µM) was incubated for 1 h at 25°C with thrombin in AB to separate GST from Q62, and then incubated for a subsequent 16 h with agitation to generate fibers. Q62 fibers were recovered by centrifugation and resuspended at 5 µM. “Capped” NM fibers (Figure 13A) were generated by incubating preformed NM fibers (2.5 µM monomer) with NM-his (5 µM), and the seeding reaction was allowed to proceed until 50% of NM-his had been converted to amyloid. This was verified empirically by determining the amount of SDS-resistant NM-his by quantitative immunoblot using an anti-Penta-His antibody in comparison to known quantities of NM-his. Fibers were recovered by centrifugation and washed (without resuspending the pellet) prior to disassembly reactions. Capped α-syn fibers were generated in the same way.

Assembled NM, α-syn, or Q62 fibers (0.5–2.5 µM monomer) were then incubated in AB with the indicated components and times (refer to figure legends). ATP was added (5 mM) plus an ATP regeneration system comprising creatine phosphate (40 mM) and creatine kinase (0.5 µM). For long-term incubations (Figures 12–14), reactions were conducted in AB supplemented with sodium azide (0.001%) and protease inhibitors (Complete, Roche). Sodium azide and protease inhibitors were removed by dialysis prior to transformation into yeast cells. For short-term incubations (Figure 11), sodium azide and protease inhibitors were omitted. Immunoblot analysis confirmed that for long-term incubations the total amount of NM or α-syn remained constant throughout the incubation. Fiber disassembly was assessed by ThT fluorescence, electron microscopy, or by sedimentation analysis (436,000 g for 10 min at 25°C) followed by determination of the amount of SDS-soluble protein in the supernatant or the amount of protein in the pellet fraction by quantitative immunoblot [49],[54],[55],[61]. For disassembly of “capped” NM or α-syn fibers, an anti-Penta-His antibody (Qiagen) was used to detect the his-tagged protein, which migrates slower than untagged protein by SDS-PAGE. Thus, untagged and his-tagged protein could be readily distinguished and quantified in comparison to know amounts of untagged or his-tagged protein.

### Disaggregation of Heat-Denatured Firefly Luciferase

Luciferase reactivation was performed as described [33]. Briefly, aggregation of firefly luciferase was elicited by heating at 45°C for 15 min in the absence or presence of indicated concentrations of Hsp26 or Hsp42. Aggregates were then incubated in the presence of Hsp104, Ssa1, and Ydj1. Luciferase reactivation was assessed using the luciferase assay system (Promega).

### Acrylodan and Pyrene Fluorescence

Acrylodan and pyrene fluorescence were measured as described [40].

### Thermal Stability of NM Fibers

The thermal stability of NM fibers (Figure 10A) was determined by incubation of fibers at increasing temperatures (25°C to 100°C in 10°C intervals) for 5 min in 1.6% SDS, followed by SDS–PAGE and quantitative immunoblot to determine the amount of SDS-soluble NM [44].

### Protein Transformation

Yeast cells from a W303-derived strain (*MATα leu2-3, -112 his3-11 trp1-1 ura3-1 ade1-14 can1-100* [*rnq* ^−^] [*psi* ^−^] [*ure-o*]) that contained an *ADE1* nonsense mutation suppressible by [*PSI^+^*] were transformed with the indicated NM or Sup35 conformers and a *URA3* plasmid. The proportion of Ura^+^ transformants that acquired [*PSI^+^*] was determined as described [44],[49]. For transformations into [*psi* ^−^] yeast cells expressing high levels of the indicated combination of Hsp26, Hsp42, Sis1, and Ydj1, a *HIS3* or *LEU2* plasmid was utilized.

### [*PSI^+^*] Induction

Δ*hsp26*, *Δhsp42*, or *Δhsp26Δhsp42* yeast strains were as described [33]. Yeast cells from a W303-derived strain (*MATa leu2-3, -112 his3-11 trp1-1 ura3-1 ade1-14 can1-100* [*RNQ^+^*] [*psi* ^−^]) were transformed with plasmids for the overexpression of Hsp26, Hsp42, Ydj1, and Sis1 together with a plasmid for the overexpression of NM fused to the yellow fluorescent protein (NM-YFP) or Sup35. All the chaperone constructs were in 2 micron plasmids under the control of the constitutive GPD promoter for high expression. The NM-YFP or Sup35 construct was under the control of the inducible Gal1 promoter. Four colonies of each of the transformants were restreaked on fresh selective plates. Only colonies that presented the correct color for [*psi* ^−^], [*PIN* ^+^] cells (i.e., red colonies) were chosen. For each [*PSI* ^+^]-induction experiment at least three independent transformants were incubated in three replicates each in 3 ml of selective liquid medium containing glucose as the sole carbon source overnight. The next day, the yeast cells were washed three times with sterile water before transferring them to selective liquid media containing galactose as the sole carbon source. The cells were incubated in the galactose media for 4 h (for NM-YFP) or 16 h (for Sup35) at 30°C before they were diluted to an OD600 of 0.002 and 80 µl of these diluted cultures were evenly plated on 25% YPD plates. The plates were then incubated for 3 d at 30°C followed by an overnight incubation at 4°C for better color development. [*PSI* ^+^] induction was scored as the number of white and pink ([*PSI* ^+^] colonies) ADE^+^ colonies divided by the total number of colonies.

### Microscopy

Three independent yeast transformants expressing the indicated chaperones together with NM-YFP were incubated overnight in liquid selective media containing glucose as the sole carbon source. The next day, the cells were recovered and washed three times with sterile water and then transferred to selective liquid media containing galactose as the sole carbon source. Cells were incubated for 4 h at 30°C and then inspected by fluorescence microscopy using a Nikon Eclipse 300 microscope with the appropriate filters.

### [*PSI^+^*] Curing

Yeast cells from a W303-derived strain (*MATa leu2-3, -112 his3-11 trp1-1 ura3-1 ade1-14 can1-100* [*rnq* ^−^] [*PSI^+^*]) were transformed with plasmids for the overexpression of Hsp26, Hsp42, or Hsp104. All these constructs were in 2 micron plasmids under the expression control of the constitutive GPD promoter for high expression. Four colonies of each of the transformants were restreaked on fresh selective plates. Only colonies that presented the correct color for [*PSI* ^+^] cells (i.e., white colonies) were chosen. For each [*PSI* ^+^]-curing experiment at least three independent transformants were incubated in three replicates each in 3 ml selective liquid medium containing glucose as the sole carbon source overnight. The next day, the cultures were diluted to an OD600 of 0.2 and incubated for 6 h. The yeast cultures were then diluted to an OD600 of 0.002 and 80 µl of these diluted cultures were spread on 25% YPD plates (resulting in ∼700 colonies per plate). The plates were then incubated for 3 d at 30°C followed by an overnight incubation at 4°C for better color development. [*PSI* ^+^] curing was scored as the proportion of red ade^−^ [*psi* ^−^] colonies.

## Abbreviations

AB: assembly buffer
α-syn: α-synuclein
BMB: 1, 4-bis-maleimidobutane
BSA: bovine serum albumin
GDH: glutamate dehydrogenase
GPD: glyceraldehyde-3-phosphate dehydrogenase
sHsp: small heat shock protein
ThT: Thioflavin-T
YFP: yellow fluorescent protein
